# Quantitative Models of the Dose-Response and Time Course of Inhalational Anthrax in Humans

**DOI:** 10.1371/journal.ppat.1003555

**Published:** 2013-08-15

**Authors:** Damon J. A. Toth, Adi V. Gundlapalli, Wiley A. Schell, Kenneth Bulmahn, Thomas E. Walton, Christopher W. Woods, Catherine Coghill, Frank Gallegos, Matthew H. Samore, Frederick R. Adler

**Affiliations:** 1 Department of Internal Medicine, University of Utah, Salt Lake City, Utah, United States of America; 2 Department of Mathematics, University of Utah, Salt Lake City, Utah, United States of America; 3 VA Salt Lake City Health Care System, Salt Lake City, Utah, United States of America; 4 Department of Pathology, University of Utah, Salt Lake City, Utah, United States of America; 5 Department of Biomedical Informatics, University of Utah, Salt Lake City, Utah, United States of America; 6 Division of Infectious Diseases, Department of Medicine, Duke University, Durham, North Carolina, United States of America; 7 Independent Risk Assessment Contractor, Idaho Falls, Idaho, United States of America; 8 Centers for Epidemiology and Animal Health, United States Department of Agriculture, Animal and Plant Health Inspection Service, Veterinary Services, Fort Collins, Colorado, United States of America; 9 Independent Risk Assessment Contractor, Santa Fe, New Mexico, United States of America; 10 Department of Biology, University of Utah, Salt Lake City, Utah, United States of America; University of Texas at Austin, United States of America

## Abstract

Anthrax poses a community health risk due to accidental or intentional aerosol release. Reliable quantitative dose-response analyses are required to estimate the magnitude and timeline of potential consequences and the effect of public health intervention strategies under specific scenarios. Analyses of available data from exposures and infections of humans and non-human primates are often contradictory. We review existing quantitative inhalational anthrax dose-response models in light of criteria we propose for a model to be useful and defensible. To satisfy these criteria, we extend an existing mechanistic competing-risks model to create a novel Exposure–Infection–Symptomatic illness–Death (EISD) model and use experimental non-human primate data and human epidemiological data to optimize parameter values. The best fit to these data leads to estimates of a dose leading to infection in 50% of susceptible humans (ID_50_) of 11,000 spores (95% confidence interval 7,200–17,000), ID_10_ of 1,700 (1,100–2,600), and ID_1_ of 160 (100–250). These estimates suggest that use of a threshold to human infection of 600 spores (as suggested in the literature) underestimates the infectivity of low doses, while an existing estimate of a 1% infection rate for a single spore overestimates low dose infectivity. We estimate the median time from exposure to onset of symptoms (incubation period) among untreated cases to be 9.9 days (7.7–13.1) for exposure to ID_50_, 11.8 days (9.5–15.0) for ID_10_, and 12.1 days (9.9–15.3) for ID_1_. Our model is the first to provide incubation period estimates that are independently consistent with data from the largest known human outbreak. This model refines previous estimates of the distribution of early onset cases after a release and provides support for the recommended 60-day course of prophylactic antibiotic treatment for individuals exposed to low doses.

## Author Summary

Anthrax poses a potential community health risk due to accidental or intentional aerosol release. We address the need for a transparent and defensible quantitative dose-response model for inhalational anthrax that is useful for risk assessors in estimating the magnitude and timeline of potential public health consequences should a release occur. Our synthesis of relevant data and previous modeling efforts identifies areas of improvement among many commonly cited dose-response models and estimates. To address those deficiencies, we provide a new model that is based on clear, transparent assumptions and published data from human and non-human primate exposures. Our resulting estimates provide important insight into the infectivity to humans of low inhaled doses of anthrax spores and the timeline of infections after an exposure event. These insights are critical to assessment of the impacts of delays in responding to a large scale aerosol release, as well as the recommended course of antibiotic administration to those potentially exposed.

## Introduction

The causative microorganism of anthrax, *Bacillus anthracis* (*B. anthracis*), is classified by the US Centers for Disease Control and Prevention (CDC) as a Category A (highest priority) bioterrorism pathogen, with the potential for causing a large number of infections and deaths after an effective aerosol release in a community [1], [2]. Reports of natural infections [3]–[6] and large scale accidental or intentional releases causing infections [7], [8] provide limited insight into the risk. To evaluate the threat posed by potential release scenarios, risk assessors, public health analysts, biodefense modelers, and other researchers require robust quantitative dose-response analyses to estimate the magnitude and timeline of potential consequences and the effect of public health intervention strategies [9]–[11], such as the administration of prophylactic antibiotic regimens to potentially exposed cases [12], and to interpret the significance of sampling results for detecting *B. anthracis* spores in indoor environments [13], [14]. For these analyses, it is particularly important to estimate the probability of infection after low dose exposures, which could cause the majority of cases after a large-scale release [15], [16].

Due to the deadly nature of the disease, there are no experimental data on exposure and response of humans to aerosolized *B. anthracis.* Analyses of quantitative information from natural and accidental exposures and infections of humans [15], [17] and experimental infections of non-human primates [18], [19] are scattered in the literature, poorly understood, and often contradictory. Mathematical dose-response modeling is useful when experimental data on the effects of low dose inhalational exposures are scarce or non-existent. These models utilize information about the height and shape of a dose-response curve at higher doses where data or estimates are available and use an assumed functional form to extend the curve to lower doses where data are not available, but where risk estimates are required. Different model forms can lead to very different extrapolated estimates from the same set of data. This creates substantial uncertainty regarding the minimum dose required to cause infection in humans [20] and the dose-dependent time from exposure to appearance of illness (incubation period), key parameters required for sound risk assessment by public health and emergency preparedness authorities [12], [21].

In this study, we critically evaluate the available published literature and identify candidate raw data sets to develop refined quantitative dose-response models for *B. anthracis* infection in humans with an emphasis on the low-dose effect. We use the resulting models to estimate the incubation period as a function of the exposure and the relationships between duration of antimicrobial treatment after exposure and the probability of infection.

## Results

### Focused review of studies on inhalational anthrax dose-response

Three outbreaks of inhalational anthrax in humans having information to estimate dose-response are the 2001 letter attacks through the U.S. Postal Service, industrial workers handling contaminated animal products in the early-mid 1900's, and an accidental airborne release of spores from a facility in Sverdlovsk, Russia in 1979.

The doses to which victims of the 2001 letters were exposed are not known, and it is a challenge to estimate exposure amounts without knowing the means by which spores were released from the envelopes, aerosolized, and inhaled. Therefore, despite modeling efforts [16], [22], these incidents shed limited information on quantitative dose-response.

Some quantitative data exist for exposure of non-vaccinated industrial workers handling animal products contaminated with *B. anthracis*. This evidence suggests that the infection rate for humans exposed in this setting is very low, especially for inhalational anthrax, as most of the infections that did occur were cutaneous [3]. Workers in one mill were thought to have been inhaling hundreds of spores on a daily basis with not a single infection documented [23]. A recent analysis of this case concluded that 600 spores or fewer would not be expected to cause disease in healthy humans and advocated the use of 600 spores as a threshold to use in risk assessments [17]. However, it is possible that the industrial workers were immune to clinical infection from repeated low-level exposure, that there were undiagnosed cases, or that infections would result from low-dose exposures of individuals with unusual susceptibility [24].

*B. anthracis* spores were accidentally released from a facility in Sverdlovsk (Russia) in the former Soviet Union in 1979, causing infections in both humans and animals downwind of the facility [7]. Doses inhaled by the infected individuals are not known, nor is it known how many spores were released from the facility. However, human dose-response information has been inferred using atmospheric data on the day the release likely occurred, the likely locations of the infected individuals when they were exposed, and the epidemiology of the tabulated cases. Meselson *et al.* [7] calculated that the attack rate at a ceramics factory 2.8 km downwind of the Sverdlovsk release was approximately 1–2% (18 out of about 1500 employees were infected, including 10 out of 450 employees working in a single unpartitioned building). Wilkening [15] analyzed the Sverdlovsk case data and applied a series of theoretical dose-response models, finding that both the spatial (distance from release) and temporal (incubation period, assumed to vary with dose) distribution of cases are consistent with dose-response curves that predict a slow decrease in the probability of infection as the dose decreases. They conclude that these data do not support a distinct exposure threshold below which no one is infected and above which everyone is infected.

In the absence of other human data, experimental studies involving non-human primates provide the best available data from which to gain insights into potentially appropriate dose-response relationships for humans. We summarize three candidate data sets and dose-response models that have been applied to them. Note that, while these studies generally use death as an endpoint and express their results in terms of lethal dose (LD), we make the assumption that LD and ID are equivalent, i.e., that infection with inhalational anthrax invariably leads to death in the absence of treatment. Two of the following three studies do not make note of infected animals that survived. The third study found evidence of infection in two surviving animals sacrificed at the termination of an experiment, but noted that “these animals were undoubtedly in the early stage of disease and presumably would have developed systemic disease and died, had the experiment not been terminated” [24]. There is also evidence that humans with inhalational anthrax infection have a fatality rate approaching 100% in the absence of treatment. Holty *et al.*, in reviewing 82 of the best-documented human inhalational anthrax cases [25], found only one instance of an infected and untreated person (an at-risk veterinarian thought to have some prior immunity) who did not progress to the fulminant stage of disease. They found only two cases (3%) of humans surviving the fulminant stage of disease under any circumstance, and both of those cases received treatment.

Glassman [26] reports on data from unpublished work performed by Jemski in which 1,236 cynomolgus monkeys (*Macaca fascicularis*) were exposed to aerosols of *B. anthracis*. While the raw data are not published, the paper reports that a log-probit model was fit to the data, resulting in a dose that is lethal to 50% of animals exposed (LD_50_) of 4,130 spores (95% confidence interval 1,980 to 8,630) and a probit slope of 0.669 probits per base-ten log dose (95% confidence interval 0.520 to 0.818). Under our definition of the log-probit model (see *Materials and Methods*), the best fit parameters are ID_50_ = 4,130 and *m* = 0.291 (Table 1, model **J**). Extrapolation using these values results in ID_10_ of 50 spores and ID_1_ of 1 spore. Without raw data, it cannot be determined whether any of the monkeys in the Jemski experiments were exposed to low doses and, if so, whether any of those doses proved fatal. Furthermore, without the full data set it is not possible to evaluate whether alternative dose-response models would have fit the data better than the log-probit model, which has been outperformed by other models in fitting other data sets [18]. Two studies [11], [15] applied a log-probit model based on the Jemski data to analyses of human exposure scenarios, although they applied ID_50_ = 8,600 (the upper limit of the 95% confidence interval reported by Glassman).

**Table 1:** Summary of anthrax dose-response models.

							Criteria satisfied			
Model	Form	Parameter values	ID_50_	ID_10_	ID_1_	ID_0.1_	1	2	3	4
*With parameter values based on Jemski (* ***J*** *) non-human primate data:*										
**J** [26] a	Log-probit	ID_50_ = 4,130; *m* = 0.291	4,130	50	1	0.1	✓	✓		
*With parameter values based on Druett (* ***D*** *)* et al. *non-human primate data:*										
**D1** [27]	Log-probit	ID_50_ = 53,000; *m* = 1.39	53,000	21,000	9,900	5,700	✓			
**D2** [18]	Exponential	*r* = 7.16×10^−6^	96,800	14,700	1,400	140	✓	✓	✓	
**D3**: our result	Exponential	*r* = 1.43×10^−5^	48,000	7,400	700	70	✓	✓	✓	
*With parameter values based on Brachman (* ***B*** *)* et al. *non-human primate data:*										
**B1** [18]	Exponential	*r* = 2.6×10^−5^	27,000	4,100	390	38	✓	✓	✓	
**B2** [35] b	Exponential (time-dep)	*r* = 4.0×10^−5^	18,000	2,700	250	25	✓	✓	✓	
**B3** [35] b	Exponential (extended)	*r* = 1.87×10^−5^; *α* = 0.9	16,000	2,800	330	41	✓	✓	✓	
**B4**: our result	Exponential (time-dep)	*r* = 6.4×10^−5^	11,000	1,700	160	16	✓	✓	✓	✓
*With parameter values based on expert (* ***E*** *) opinion:*										
**E1** [31] c	Log-probit	ID_50_ = 8,940; *m* = 0.621	8,940	1,135	211	62				
**E2** [9]	Age-dependent log-probit	see *Materials and Methods*	8,400	1,500	280	86				
**E3** [10]	Age-dependent linear	see *Materials and Methods*	8,700	1,300	130	13		✓		
**E4** [16]	Age-dependent logit	see *Materials and Methods*	8,300	1,500	210	22		✓		
**E5** [32] d	Exponential (time-dep)	*r* = 8.1×10^−5^	8,600	1,300	120	12		✓	✓	✓

Model formulas and parameter value definitions are described in detail in the Materials and Methods section. ID estimates for age-dependent models rely on estimates of the age distribution of the United States population from the 2010 census. Criteria used to evaluate the models are 1) the parameter values are derived from dose-response data; 2) the shape of the dose-response curve is consistent with Sverdlovsk data; 3) the model is derived from mechanistic assumptions; and 4) the model estimates the incubation period.

a Papers [11], [15] citing model **J** instead used ID_50_ = 8,600, which is just within the 95% confidence limits reported in [26].

b Models **B2** and **B3** estimate the time from exposure to infection take-off and death, but not the incubation period (time from exposure to onset of symptoms).

c Papers [11], [15] citing model **E1** instead used ID_50_ = 8,600, which is within the range reported in the original paper.

d For model **E5**, the original paper [32] estimated the time-dependent parameter *θ* from data and did not specify an estimate for *r*, but papers applying this model [11], [15] used the *r* value given above under an assumption of ID_50_ = 8,600, comparable to other models based on expert opinion.

Two studies contain raw data from a substantial number of monkeys exposed to a range of dose amounts. Druett *et al.* [27] exposed rhesus monkeys (*Macaca mulatta*) to aerosols of *B. anthracis* spores resulting in a range of inhaled doses estimated between about 35,000 to 200,000 spores. We summarize the data from these experiments in Table S1. The authors also fit a log-probit model to their data (Table 1, model **D1**) resulting in optimal parameters equivalent to ID_50_ = 53,000 spores (95% confidence interval 30,000 to 52,000) and *m* = 1.39. Haas [18] reported a fit of the exponential model (model **D2**) to this data set and also stated that the best fit log-probit and beta Poisson models did not provide a statistically significantly improved fit compared to the exponential model.

The second study containing raw data, Brachman *et al.* [24], exposed cynomolgus monkeys to *B. anthracis*-contaminated air from a goat hair mill. The data consist of estimated dosage and the number of deaths from anthrax, sacrifice, or other cause on each day across three model runs and are shown graphically in [24]. We visually estimated the daily exposure data from their figures and manually adjusted those estimates until they were consistent with the cumulative dose numbers reported in the source text. Our estimates of these numerical data are shown in Tables S2, S3, S4. The authors did not fit a dose response model to their data, but two more recent studies have done so. Haas [18] used an averaging technique [28] to fit a time-independent exponential model (Table 1, model **B1**) to the data, and Mayer *et al.* fit a time-dependent exponential model and an extended exponential model (Table 1, models **B2** and **B3**).

The published literature also includes quantitative human inhalational anthrax dose-response estimates based on the opinion or judgment of experts. For example, biodefense experts from the US Army Institute of Infectious Diseases (USAMRIID, Fort Detrick, MD) state the infective dose (presumably ID_50_) of inhalational anthrax for humans is 8,000–50,000 spores [29], [30]. An expert elicitation of seven anthrax subject matter experts [31] indicated an ID_10_ of 1,000–2,000 spores, an ID_50_ of 8,000–10,000 spores, and an ID_90_ of 50,000–100,000 spores. Webb and Blaser [16] extended those expert-derived estimates to age-specific values for the ID_10_ and ID_50_, but without providing quantitative evidence or reasoning used to derive these estimates. Several dose response models have been proposed and applied based entirely or in part on the values from these expert elicitations (Table 1, models **E1**–**E5**).

### Evaluation of published dose-response models for human exposure to *B. anthracis*

We evaluate the previously published models against the criteria listed in *Materials and Methods* in Table 1. Versions of six of the models in Table 1 (**J** and **E1**–**E5**) have been applied in recent mathematical modeling or simulation studies of human exposure to anthrax [9]–[11], [15], [16], [32]. Models **J**, **D1**, **D2** and **B1**–**B3** were fit to one of three non-human primate dose-response data sets and, therefore, satisfy criterion 1 (although model **J** is based on a data set by Jemski for which the raw data are not published, which limits transparency). Models **E1**–**E5** do not have a clear basis in quantitative dose-response data, but are instead based entirely or partly on assumptions, recommendations, or expert opinions for which the reasoning has not been made clear in published accounts. All models except for three of the log-probit models with steeper slopes (**E1**, **E2**, and **D1**) produce dose-response curves with shapes that either were shown to be consistent with the Sverdlovsk data in Wilkening [15] or produce similar estimates to the models tested in that study and, therefore, satisfy criterion 2. The models taking the exponential form (**E5**, **D2**, and **B1**–**B3**) are based on simple assumptions about the fate of individual spores inhaled in the lung, satisfying criterion 3, while the other models make use of statistical distributions with no clear basis in assumed mechanisms of infection. Model **E5** produces incubation period estimates as an extension of the assumptions that form the basis of the model and, therefore, satisfy criterion 4. Models **B2** and **B3** produce estimates for the time course of infection but not for the incubation period. I.e., they specify time to infection take-off (initial germination of inhaled spores) and to death, but not to onset of symptoms. The other previously existing models do not contain time components for disease progression among those infected. Although an incubation period distribution can be added to any dose-response model exogenously (as was done by Wilkening [15] to a version of model **J** and model **E2**), our preference under criterion 4 is for models in which the incubation period estimates are derived *ab initio* in conjunction with a dose-response model.

Of the five models with a quantitative basis in expert opinion, model **E5** has the most (three) of the desired characteristics of an anthrax dose-response model. However, while some of the time-based parameters of this model have been estimated from non-human primate data and human data from Sverdlovsk [32], the full dose-response model is incomplete without assuming a fixed point on the dose-response curve (e.g., the ID_50_) which does not have a firm basis in those data. Non-human primate data sets can be used to fill that need. Model **J** based on the Jemski data does not satisfy criteria 3 and 4, and the raw data are not available to attempt further modeling with improved characteristics. Therefore, we focus on models fit the Druett *et al.* and Brachman *et al.* data sets in the following sections.

### Model fitting to the Druett *et al.* data set

We checked the results for the optimal parameters of the log-probit model **D1** and the exponential model **D2** when fit to the Druett *et al.* data. Our best fit parameters for the log-probit model confirm the results of model **D1**. For the exponential model, our best fit parameter is *r* = 1.43×10^−5^ (95% confidence interval 0.92×10^−5^ to 2.19×10^−5^), which is twice the estimate of model **D2**. We have listed our novel result as model **D3**, and we explain the source of the difference from model **D2** below. We also fit the beta Poisson model to the data set, and the result produced a nearly identical curve to model **D3**, so we did not list it in Table 1. The exponential model contains one fewer parameter than the beta Poisson model and is, therefore, more parsimonious, so the beta Poisson model need not be considered further, as it does not improve the fit to the data.

Models **D1** and **D3** have a statistical deviance (defined in *Materials and Methods*) of and 10.3 and 11.3, respectively, which are less than the corresponding 95^th^ percentile chi-squared statistics (14.1 and 15.5) with degrees of freedom equal to the number of dose points (9) minus the number of parameters in each model (2 and 1). Under this criterion, both models provide an adequate fit to the data [33]. The deviance under model **D1** is lower than under **D3**, which suggests a better fit, but the difference is less than the difference in the chi-squared statistics, so that the exponential model would be chosen as the best combination of fit and parsimony [33].

The ID estimates shown for models **D1** and **D3** in Table 1 illustrate the sensitivity of extrapolated estimates to model choice. The ID_50_ estimates, which are within the range of the doses actually supplied to the animals, agree closely, whereas the estimates for doses farther below the lowest dose from the data set (≈35,000 spores) differ substantially. While the extrapolations from the exponential model are better supported according to the statistical criteria described above, even a small amount of additional data at lower doses could have shifted support to the estimates of the log-probit model.

Dose-response models fit to the Druett *et al.* data have not been applied to mathematical models or simulations of human anthrax exposure, to our knowledge. While both the exponential and log-probit models provide adequate fits to the data and, therefore, satisfy our first criterion, the exponential model better satisfies our other criteria: it is derived from testable, mechanistic assumptions, while the log-probit model is not [18], and it produces a less steep dose-response curve that is more consistent with the Sverdlovsk data [15]. However, neither model can satisfy our criterion of providing a time-to-infection component without making additional unsupported assumptions, as the time of death was not reported in the Druett *et al.* data. Therefore, we turn to the Brachman *et al.* data, which have the ability to support a model that satisfies all four of our criteria.

### Estimates of infectious doses and time course of infection using Brachman *et al.* data

We fit a novel Exposure–Infection–Symptomatic illness–Death (EISD) model to the Brachman *et al.* data set [24], resulting in Model **B4** (Table 1). The overall model, summarized here and described in detail in *Materials and Methods*, contains five parameters. The exponential dose-response model parameter *r*, the probability of one spore germinating before being cleared, governs the probability that infection will eventually occur after exposure to a given dose. Among those infected, the time from exposure to infection, defined as the time of the first successful spore germination leading to a sustained population of vegetative cells in the host, is governed by the parameters *r* and *θ*, the rate of clearance of spores from the lung. The time from infection to the onset of symptomatic illness is represented by the fixed parameter *T*, and the time from the onset of symptoms to death is governed by the parameters *a* and *b*, which are shape and scale parameters of a gamma distribution.

Estimates for three of these five model parameters are available from independent data of *B. anthracis* infections in humans and in non-human primates. Brookmeyer *et al.* [32] calculated the probability-per-time for clearance of spores from the lung, *θ*, to be 0.07 per day, based on data from examination of the lungs of non-human primates at varying times after inhalation [34]. Data are also available for the time between the onset of symptoms and death in humans. Holty et al. [25] assembled data from 82 human inhalational anthrax cases, occurring between 1900 and 2001, that met their inclusion criteria concerning sufficient documentation of anthrax infection, symptoms, and treatment. Their data set includes, for 75 of the cases, the time from the onset of symptoms to death, if it occurred, and/or to appropriate antibiotic therapy, if it occurred, which may have prevented or delayed death. We used a maximum likelihood procedure, designed to account for time censoring (see *Materials and Methods*), to fit a gamma distribution for the time between onset of symptoms and death to their compiled data set. We determined the shape parameter *a* = 5.43 and scale parameter *b* = 0.864, which results in an average time of 4.7 days, with a standard deviation of 2.0 days.

By fixing those values of the three parameters *θ* (rate of clearance of spores from the lung), *a* (shape parameter), and *b* (scale parameter), we estimated the values for the remaining parameters *r* (probability of one spore germinating before being cleared) and *T* (delay between initial spore germination and onset of symptoms) from the Brachman data. The best fit model estimates *r* = 6.4×10^−5^ (95% confidence interval of 4.0×10^−5^–9.5×10^−5^) and *T* = 2.3 days (0–5.4). The optimal deviance of 129 is less than the corresponding 95^th^ percentile chi-squared statistic (170) with 142 degrees of freedom (the number of daily dose points minus the number of optimized parameters in the model), suggesting that the model provides an adequate fit to the data. The optimal value of *r* leads to an ID_50_ of 11,000 spores (7,200–17,000), ID_10_ of 1,700 spores (1,100–2,600) and ID_1_ of 160 spores (100–250). The optimal value of *T*, when combined with the dose-dependent delay from exposure to infection, produces dose-dependent incubation periods (exposure to symptom onset). For an ID_50_ dose, the median incubation period is estimated to be 9.9 days (7.7–13.1). For ID_10_, the estimate is 11.8 days (9.5–15.0) and for a low dose of ID_1_, the estimate is 12.1 days (9.9–15.3).

Our best fit model to the Brachman data satisfies all four criteria that we propose for a defensible anthrax dose-response model that is useful for quantitative risk assessment. All parameter values are transparently derived from human and non-human primate data, the model is derived from biological assumptions about the establishment of infection and progression of disease, the model provides estimates for dose-dependent infection probability and distribution of incubation period, and the shape of the dose-response curve is consistent with what was observed in the Sverdlovsk data. We compare the results from this model to others in the literature in the following sections.

### Comparison of best fit models to previous studies

**Figure 1:**
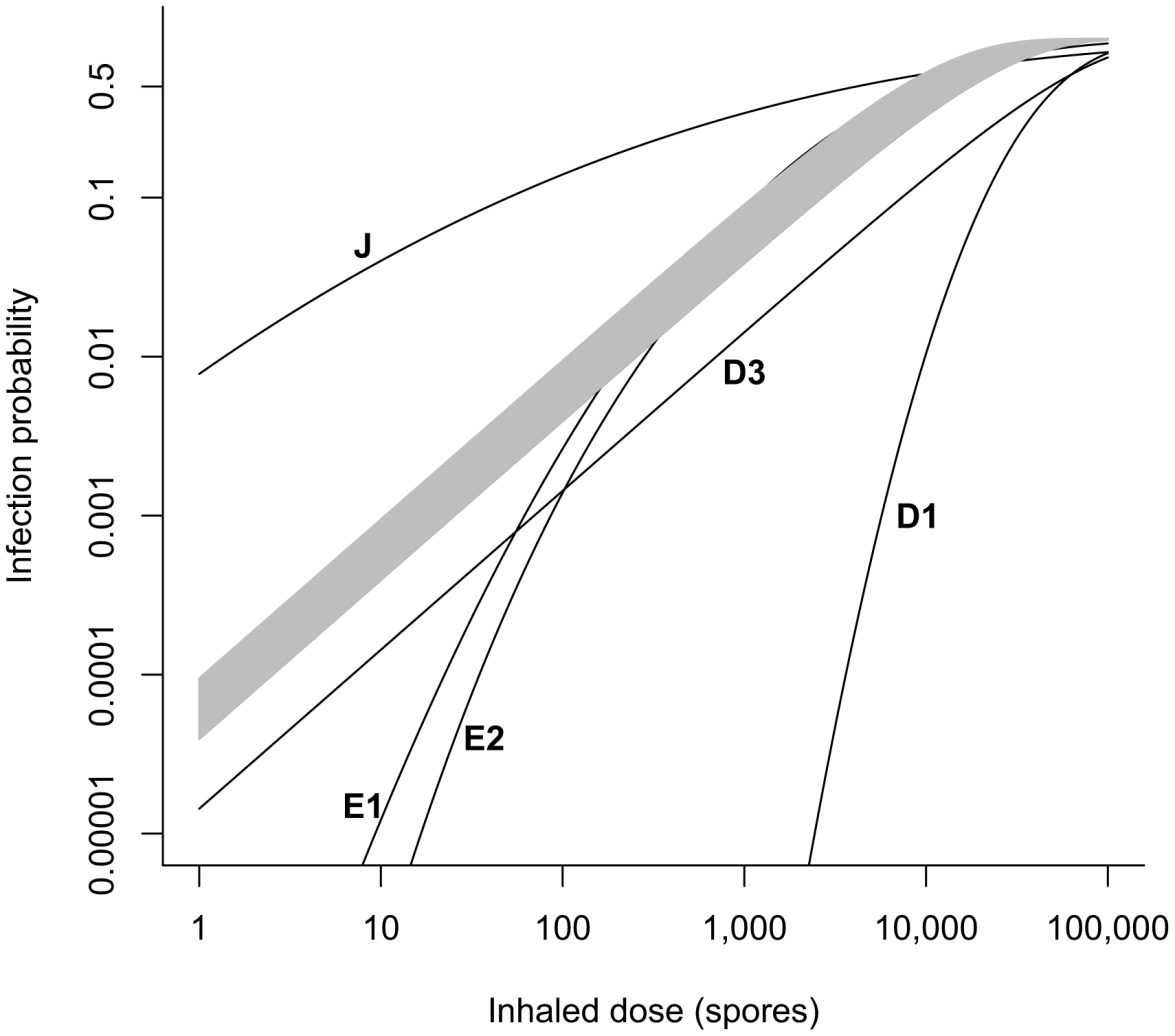
Comparison of dose-response models. Our best fit exponential model **B4** based on Brachman data (shaded region = 95% confidence range) is compared to selected other models from Table 1. Models **E3**, **E4**, and **E5** fall entirely within the shaded region. Model **B2** falls just below the lower boundary of the shaded region and is visually indistinguishable from it. We omit the curve for model **D2** in this figure, as our fit of the exponential model to the Druett *et al.* data set (**D3**) replaces the fit done by Haas (**D2**).

We compare the uncertainty range of the dose-response curve (probability of infection at any time after exposure to a given dose) produced by model **B4** to the curves from selected models shown in Table 1, focusing on low doses (Figure 1). Models **E3**, **E4**, and **E5** from Table 1 are in agreement with model **B4**, as those curves fall entirely within the shaded region representing the 95% range. Models **E1** and **E2** are in agreement for doses above 200 and 400 spores, respectively, but they produce a significantly lower probability of infection for lower doses. Model **J** produces a significantly higher infection probability at doses less than 5,000 spores. Models **D1** and **D3** produce lower infection probabilities at all doses.

Our optimal estimate for the exponential model parameter *r* fit to the Druett data set (model **D3**) is two times higher than the value calculated by Haas [18] (model **D2**) for the same model fit to the same data set. The lower infectivity produced by model **D2** results from an estimated respiration rate of 2.4 L/min rather than the value of 1.2 L/min reported and used for calculations in the original paper [27]. See Table S1 for our calculation of the doses inhaled by each group of non-human primates in the original study.

**Figure 2:**
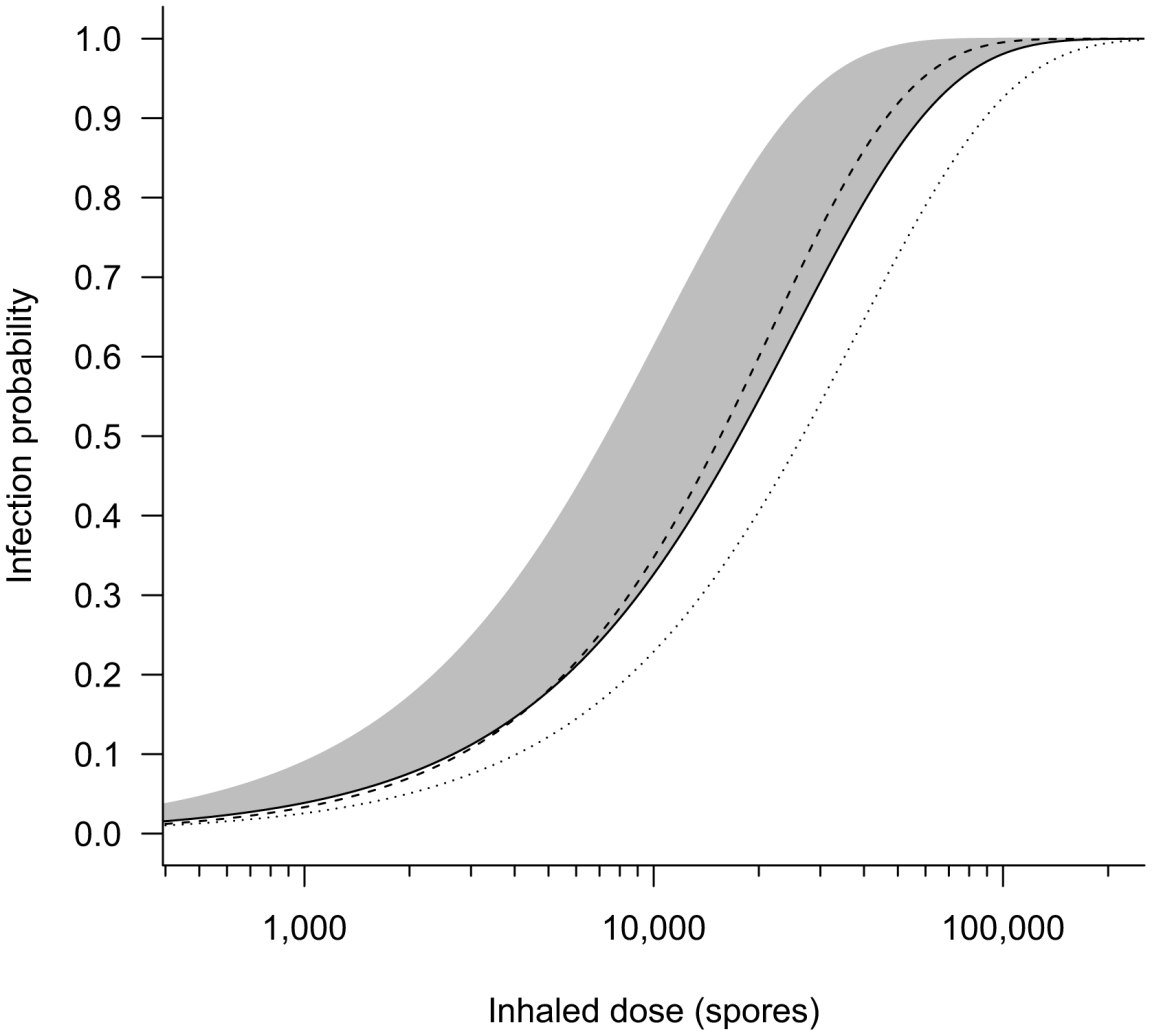
Comparison of dose-response models fit to the Brachman data. Our best fit exponential model **B4** based on the Brachman data (shaded region = 95% confidence range) is compared to other models fit to the same data set. Dashed line = Mayer *et al.* [35] extended exponential model **B3** (*α* = 0.90, *r* = 1.87×10^−5^); solid line = Mayer *et al.* [35] exponential model **B2** (*r* = 3.95×10^−5^); dotted line = Haas [18] exponential model **B1** (*r* = 2.6×10^−5^). The Haas curve would shift very close to the Mayer exponential curve if the correct cumulative dose is applied (see Table S5).

For the Brachman data, our model **B4** produces an estimated range for the exponential model parameter *r*, and thus for infectivity at a given dose, that is somewhat higher than the values calculated by Haas [18] (model **B1**) and Mayer *et al.* [35] (model **B2**) for exponential model fits to the same data set (Figure 2). There were several assumptions made by the three studies that contributed to the differing infectivity results among these three models.

In theory, the averaging technique used by model **B1** should have produced the same value as the other two models for the exponential parameter *r* [28]. An important reason why the model **B1** result is lower is that its calculation resulted from an incorrect assumption of a higher total cumulative dose for runs three and four of the Brachman experiments than was reported in the original paper (see Table S5). We recalculated *r* using the technique of model **B1** with the correct dose values and found *r* = 3.8×10^−5^, which is very close to the result of model **B2**. Also, model **B1** included animals that died of non-anthrax causes during the Brachman experiments in the group of survivors; if those cases had been excluded entirely, their estimate for *r* would have increased slightly.

The main reason why our novel result for the parameter *r* (model **B4**) is less than both model **B2** and corrected model **B1**, is that models **B1** and **B2** both assumed that all animals sacrificed and not found to be infected at the end of each run would not have become infected had the experiment continued. Our modeling process allows for the possibility that animals dying of other causes or sacrificed could have become infected with *B. anthracis* at later dates had they lived. Model **B4** estimates that there was approximately a 7%, 4%, and 4% chance of infection after the day the animals were sacrificed in Brachman runs 3, 4, and 5, respectively, assuming no further exposure. If those probabilities are accurate, then there likely would have been a few more deaths from anthrax across the three runs had the animals lived longer.

Our model **B4** also differs from model **B2** in its assumptions and results for the time course from exposure to death in anthrax cases. In their procedure for model **B2**, Mayer *et al.* [35] independently assumed that the delay between infection take-off and death was 1, 2, 3, or 4 days with equal probability (an assumption not based clearly on data). They then optimized their equivalent to our parameter *θ* to account for the remaining portion (exposure to infection take-off) of the overall delay between exposure and death, finding an optimal value of *θ* = 0.11. We chose different assumptions that rely more directly on quantitative data, fixing *θ* = 0.07 based on data of spore clearance rates in non-human primates and expressing the symptoms onset to death delay with a gamma distribution fit to rigorously reviewed human anthrax case data, leaving the infection to symptoms onset delay *T* to be optimized (resulting in *T* = 2.3 days).

**Figure 3:**
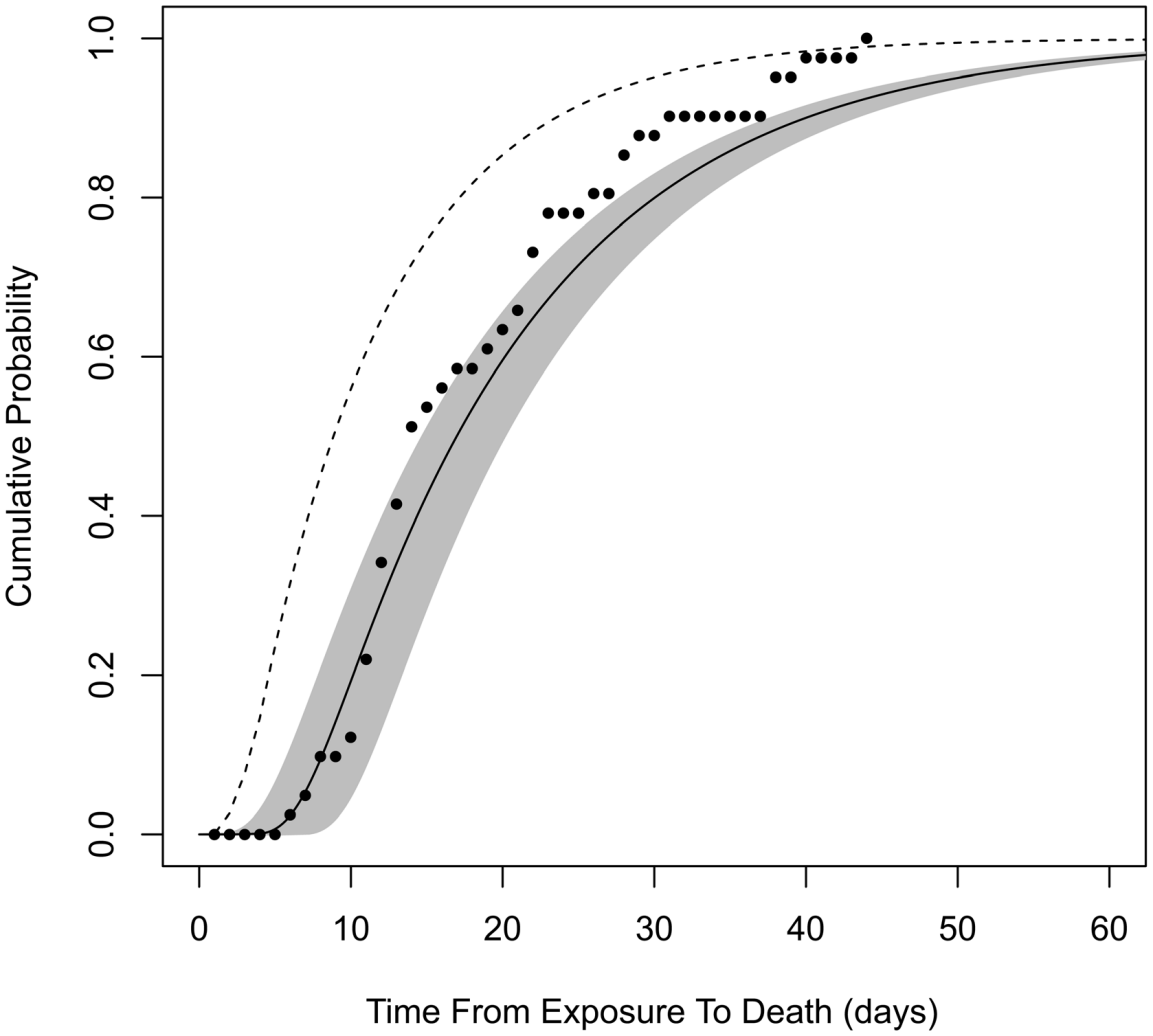
Cumulative distribution function for time from exposure to death. Assuming exposure to ID_1_, solid curve is the distribution produced by our model **B4** (shaded area is the 95% confidence region). Dashed curve is produced by model **B2**. Points are from autopsy-confirmed anthrax deaths after the Sverdlovsk release.

Model **B2** does not provide estimates of the incubation period that can be compared to our estimates from model **B4**, because model **B2** does not specify the time of symptoms onset in its formulation. However, both models do provide estimates for the time from exposure to death (the endpoint of the Brachman experiments). We find that our model **B4** produces significantly longer estimates than model **B2** for this time interval. For example, after a single ID_10_ exposure, our model **B4** estimates a median time from exposure to death, among those infected and untreated, to be 16.6 days (95% confidence range, 14.4 to 19.8 days), while model **B2** estimates 8.6 days. It is unclear why these time progression estimates differed so widely, given that the two models were fit to the same data set. We evaluated model **B2** against our optimization criterion (the minimized deviance *Y*, defined in *Materials and Methods*) and found that it provided a poorer fit to the Brachman data by our measure (*Y* = 158 compared to *Y* = 129 for our model **B4**). Our model **B4** also outperforms model **B2** in describing the distribution of human exposure-to-death time estimates from the Sverdlovsk release reported by Abramova *et al.* [36] (Figure 3).

To further explore the implications of our assumptions in constructing model **B4** compared to model **B2**, we tested the sensitivity of our results to our choice of *θ* (Figure S1). We reran our optimization procedure fixing *θ* = 0.11, which results in optimized values of *r* = 5.6×10^−5^ and *T* = 3.4 days. The new *r* value is a small decrease in the infectivity estimate compared to our model **B4** result, causing an increase in the ID_50_ estimate from 11,000 to 12,000 spores. The new value of *θ* caused the optimized time from initial germination to symptom onset to increase by about 1.1 days; however, the new value of *θ* causes the median time from exposure to initial germination to decrease by about 3.6 days at low doses. Therefore, applying *θ* = 0.11 instead of 0.07 would have decreased our median incubation time and time-to-death estimates at low doses by about 2.5 days, not enough to fully account for the 8-day difference described above between models **B4** and **B2**.

A final difference between model **B2** and **B4** is that the model **B2** parameters were only fit to runs 3 and 4 of the Brachman experiments, whereas we made use of runs 3, 4, and 5 (see Tables S2, S3, S4) in producing model **B4**. We found that deleting the data from Brachman run 5 had a negligible effect on our infectivity results (the optimal *r* value was unchanged to two significant digits), so the additional data we incorporated did not contribute to the differing infectivity results of the two models.

**Figure 4:**
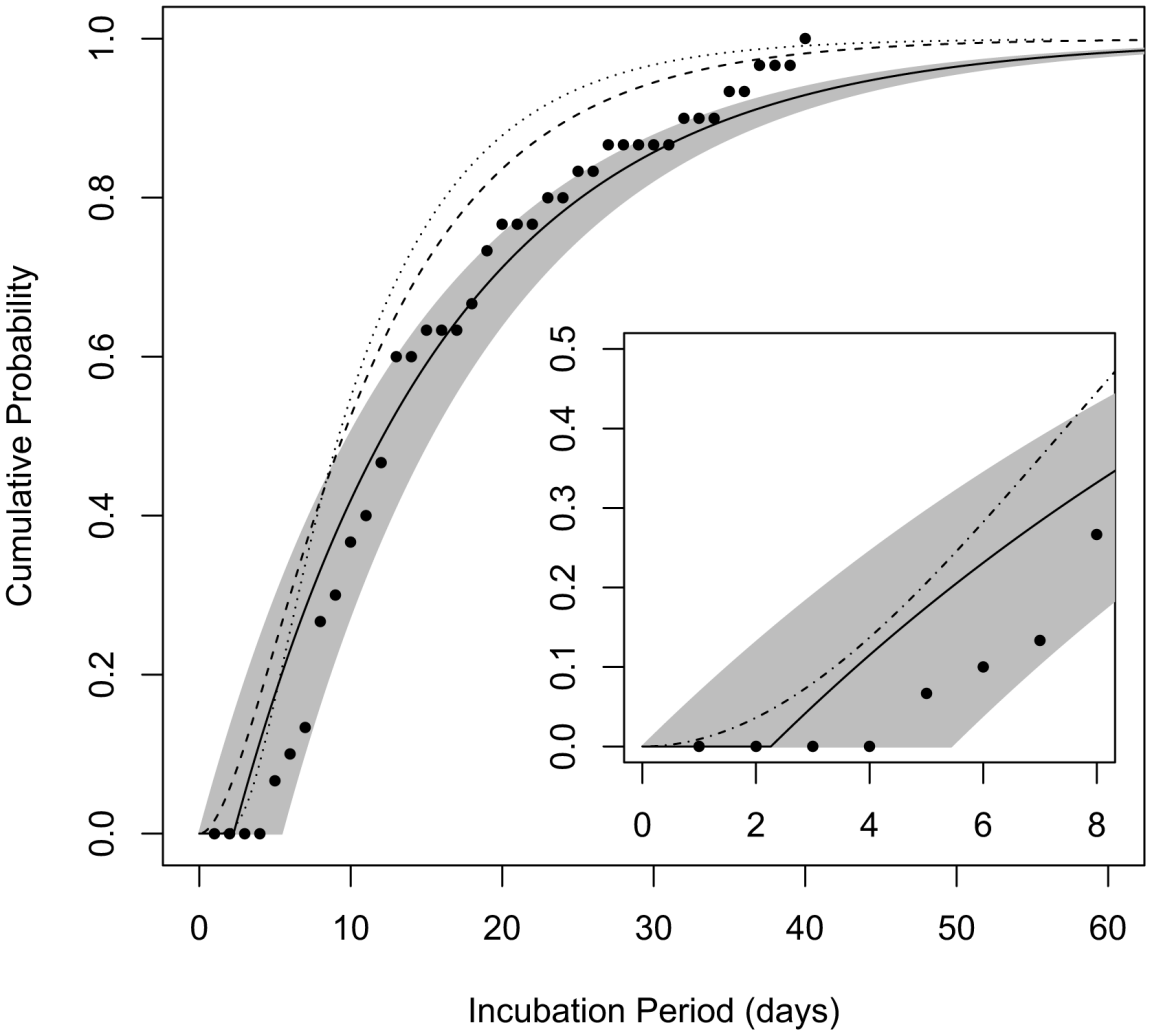
Cumulative distribution functions for the incubation period among those infected by a low dose. The curves show the probability that a given incubation period (time from exposure to symptoms) among those infected by the ID_1_ would be less than the given number of days post-exposure. Solid line represents the estimate produced by model **B4** and the shaded area spans the 95% confidence bounds; dashed line is the curve produced by the model of Brookmeyer et al. [32]; dotted line is the curve produced by the model of Wilkening [37]; points are data from 30 autopsy-confirmed anthrax cases after the Sverdlovsk release [36]. Inset: comparison of our model **B4** with the model proposed for use by the IOM [12] for the anthrax incubation period distribution over the first 8 days after exposure (dash-dotted line).

Next, we compare the incubation period distribution produced by our model **B4** to three other estimates of the incubation period for human inhalational anthrax found in the literature [12], [32], [37] (Figure 4). Our model is unique in that, while the shape of the dose-response curve being consistent with the Sverdlovsk data was a criterion for model choice, we did not actually use incubation period data or time-to-death data from Sverdlovsk to determine parameter values. Therefore, we also check our model's estimates against the Sverdlovsk data (Figure 4) as a validation for the utility of applying model **B4** to a human outbreak.

The Institute of Medicine (IOM) performed a detailed review [12] of data from analyses of Sverdlovsk patients: Abramova *et al.* [36] reported on 41 autopsy-confirmed cases, among which 30 cases had known dates of symptom onset; Meselson *et al.* [7] compiled data from 77 cases, 60 with known symptoms timing, but no additional confirmed cases beyond those that were reported by Abramova *et al.*; Brookmeyer *et al.* [38] analyzed 70 cases with known symptoms onset dates, but again, no additional cases beyond the Abramova data that were confirmed by autopsy or microbiological testing. The IOM committee reviewing these data wrote “in its analysis of previous anthrax incidents, the committee required either microbiologic or histopathologic confirmation of infection with *B. anthracis* when determining the minimum incubation period of patients with inhalational anthrax” [36]. We chose to follow the lead of this committee and used only the autopsy-confirmed Abramova *et al.* data in Figure 4 to test the performance of our model. These data, when choosing April 2, 1979 as the assumed date of release and exposure (an assumption supported by compelling evidence [12]), consist of 30 estimated incubation periods ranging from 5 to 40 days, with median 13 days and mean of 16.0 days. Of the data from the other two studies excluded from this set, the IOM cast doubt in particular on unconfirmed reports of shorter incubation periods, as low as 2 days, which are not well supported [12].

We compared the incubation period distribution provided by our model **B4** under the assumption of exposure to the ID_1_ (consistent with an approximate 1% attack rate observed at given locations downwind of the Sverdlovsk release [7]) to the distribution of the estimated incubation periods of confirmed Sverdlovsk cases (Figure 4). Our model appears to provide a good match to these data, as most points fall within our 95% confidence range, despite the fact that these data were not used in fitting parameter values for our model. We find that model **B4**'s consistency with these Sverdlovsk incubation time data is robust to assuming that infected cases were exposed to a much higher dose (ID_50_) and to the alternate assumption of *θ* = 0.11, discussed above (Figure S1).

We also compare our model to dose-dependent incubation period distributions provided by Brookmeyer *et al.* [32] and by Wilkening [37] (Figure 4) and to a dose-independent incubation period model provided by the IOM for use by risk assessors in comparing intervention strategies over the first 8 days after exposure (Figure 4 inset). Descriptions of the three models can be found in *Materials and Methods*. These models fall within the 95% confidence bounds of model **B4** during the first 7 days after exposure, but they estimate that cases after the first week would appear more rapidly than does our model. We note that the IOM model was not designed to be accurate after the first eight days. The other two models made use of the larger Sverdlovsk data sets, including incubation time estimate of cases not confirmed to the standards of the IOM review, which shows that those unconfirmed data were skewed towards earlier dates of onset.

### Estimation of the effect of antibiotic course duration on reducing the probability of infection

**Figure 5:**
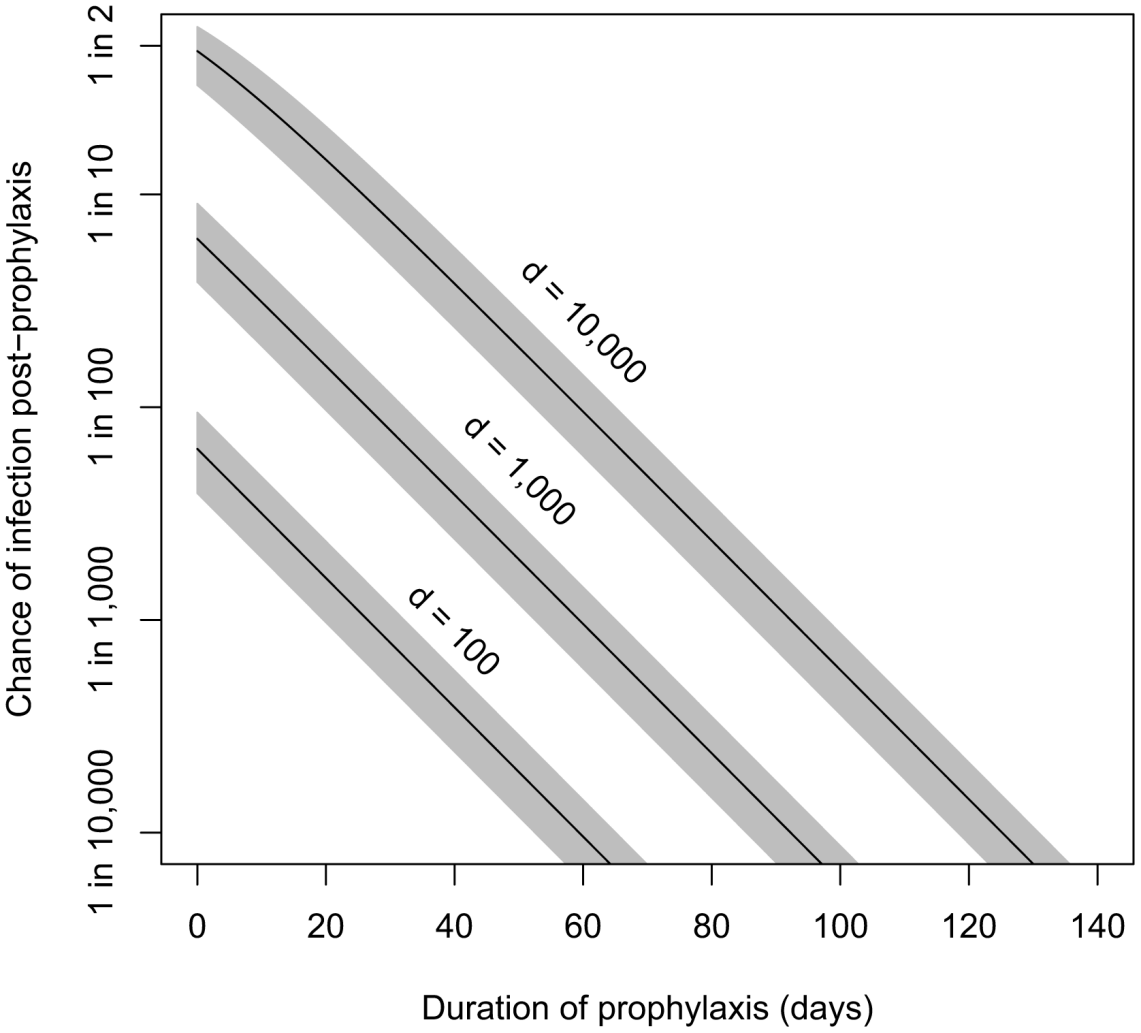
Estimated relationship between duration of prophylaxis and subsequent chance of infection. Relationship between duration of prophylaxis (days, post-exposure) and the estimated chance of infection after antibiotics are no longer taken, at doses of 100, 1,000, and 10,000 spores. We assume the probability-per-day for clearance of spores from the lung, *θ*, is 0.07, and shaded areas are the confidence regions based on the 95% confidence interval for model **B4**'s fitted parameter *r* (probability of one spore germinating before being cleared).

Our incubation period estimates assume that prophylactic treatment is not administered to the population. In the case that prophylaxis is made available, the model also can estimate the effects of various durations of antibiotic use on reducing the probability of infections. Brookmeyer *et al.* [39] provided an equation for the probability that an individual exposed at a given level and adhering to an effective prophylactic regimen would becomes infected after ending use of antibiotics a given number of days after exposure. We have reproduced their equation using our parameter definitions in *Materials and Methods*. Given that the Brookmeyer paper applied the same spore clearance rate (*θ* = 0.07) as our model **B4**, their results are applicable to our model. For example, they calculated that, to reduce the risk of infection below 0.01% (one in ten thousand chance), someone exposed to the ID_0.5_, ID_1_, ID_10_, and ID_50_ would have to remain on antibiotic prophylaxis for at least 56, 66, 99, and 126 days after exposure, respectively [39]. Our contribution to this result is that, using the Model **B4** result, we can express the exposures in terms of the number of spores in the dose in addition to the ID level (see, e.g., ID_1_, ID_10_, and ID_50_ shown in Table 1 for model **B4**). In Figure 5, we show the relationship between duration of prophylaxis (days, post-exposure) and the estimated chance under model **B4** of infection in humans after antibiotics are no longer taken, at exposures of 100, 1,000, and 10,000 anthrax spores.

A 60-day course of antibiotics for those potentially exposed has been recommended by the CDC [40]. Brookmeyer *et al.* suggested that this course should be adequate at doses lower than the ID_1_. Using model **B4**, we estimate that ending a course of antibiotics 60 days after exposure would reduce the probability of infection below 0.1% for those exposed to doses of 1,000 spores (ID_6_) or less, and below 0.01% for those exposed to doses of 100 spores (ID_0.7_) or less.

## Discussion

As with most biothreat pathogens, the dose-response relationship of aerosolized *B. anthracis* in humans, especially in the low-dose range, remains highly uncertain. In the absence of human experimental data, risk assessments have relied on dose-response models that extrapolate from information on higher doses in animals. Despite an impressive body of published literature on this topic, these models have produced contradictory results and are based on assumptions that are poorly understood. In order to make informed decisions regarding preparations for and response to accidental or malevolent release of *B. anthracis* spores, the scientific and public health community need to have access to plausible and defensible models. These models ideally should be based on available measured dose-response data from non-human primates, be derived from mechanistic assumptions, provide estimates of incubation periods, and produce plausible results when applied to human exposure scenarios.

Using our focused evaluation of the published literature on significant accidental and intentional exposures to humans and on non-human primate studies, we identify candidate dose-response models that satisfy our objective criteria and fit them to non-human primate dose-response data. We use these refined models to estimate incubation periods and evaluate the duration of antimicrobial treatment required to achieve a low probability of infection after exposure to aerosolized anthrax spores.

We propose Model **B4** (Table 1) for use in quantitative analyses that require dose-response assessment for human inhalational anthrax, because it satisfies all four of our proposed criteria and improves on existing models fit to the same data set. The ID_50_ (7,200–17,000) and ID_10_ (1,100–2,600) confidence ranges produced by model **B4** are remarkably consistent with the corresponding ranges produced by an expert panel surveyed in 1998 [31], (8,000–10,000) and (1,000–2,000), respectively. While four of the seven subject-matter experts questioned in that study reported having experience with animal testing, it is not known if or how their ID estimates were based on nonhuman primate data. Models **E3**, **E4**, and **E5** (Table 1), which were fit to these expert estimates, produce low-dose extrapolations that are consistent with those produced by our model **B4**.

At a dose of 600 spores, our model **B4** estimates that infection would occur sometime after exposure in about 2–6% of untreated cases, with the incubation time distribution of those cases being close to what is shown in Figure 4. This estimate would appear to run counter to the conclusion by Cohen *et al.* [17] that 600 spores can be used as threshold in risk analyses. For example, a risk analysis estimating a 2–6% infection rate for visitors to a contaminated building likely would not conclude that building is safe for the general public. However, these authors recommend the 600 spore threshold only for healthy individuals. A widespread release likely would include individuals who are unusually predisposed or immune-compromised, for whom exposure to 600 spores or less could result in infection. Although the exponential model we develop here does not explicitly include heterogeneous susceptibility in the host population, the estimated average susceptibility should be conservative enough to apply the model to an exposed population that includes a larger proportion of susceptible individuals. Furthermore, the bacterial strains present at the factories on which the Cohen *et al.* estimate is based may have been less virulent to humans than other strains that could be released.

It has not been proven that a single dose less than the 600 spore threshold recommended by Cohen *et al.* has ever infected a human or a non-human primate. To our knowledge, the lowest dose shown to cause infection in non-human primates occurred in the first part of run 5 in the Brachman experiments, in which two animals (8.3%) died of anthrax after inhaling an estimated cumulative dose of approximately 950 spores over three days. If it is true that infections never occur in humans at doses in the hundreds of spores, the log-probit model fit to the Druett *et al.* data set (Model **D1**, Table 1) might be a viable alternative. With this model, the estimated probability of infection at 600 spores is less than one in one billion. However, a dose-response curve with a slope as steep as Model **D1** is not consistent with the spatial distribution of human cases observed at Sverdlovsk [15]. Also, given that the exponential model **D3** provides an equally good fit to the Druett data, we find the choice of the log-probit model **D1** to be unjustified in the absence of a coherent biological theory that can explain steepness of the dose-response curve.

A model applying one such biological theory is provided by Mayer *et al.* [35], who extended the exponential model to investigate potential effects of immune system dynamics [41]–[43], using an assumption that the immune system is more likely to be overwhelmed when receiving a large dose all at once as compared to receiving the same total exposure in a series of smaller doses over an extended period. I.e., the per-spore infection probability would be higher after a higher single dose, thus producing a dose-response curve that is steeper than the exponential model, which assumes that the size of a single dose does not affect the per-spore infection probability. However, when they fit their model to the Brachman data (model **B3**), the resulting dose-response curve was only slightly different from the best fit curve under the more parsimonious exponential model (model **B2**). We also tested their model against the Druett data and found that, similar to the log-probit model **D1**, the improvement in fit over the exponential model did not justify the decrease in parsimony under the criterion we used for model comparison [33]. These results provide some justification for recommending the simpler exponential model for use in modeling and simulation studies until the role of the immune system in preventing infection at various levels and time courses of exposure is better understood at a quantitative level.

While the log-probit model **D1** discussed above produces a steep dose-response curve, the log-probit model based on the Jemski data set, model **J**, has the most gradual slope of all models found in the literature. It produces very high infectivity estimates at low doses, significantly higher than those produced by our recommended model **B4**. Wilkening [15] was unable to rule out the possibility that the shape of the model **J** dose-response curve was consistent with the spatial distribution of Sverdlovsk cases. Heterogeneity in host susceptibility could provide a biological explanation for a dose-response curve with a more gradual slope than the exponential model. That is, some individuals in a population might be significantly more susceptible to lower doses, while others may be able to tolerate high doses with unusually high probability. The beta Poisson dose-response model (see *Materials and Methods*) can quantify this kind of heterogeneity in a transparent manner that encompasses the mechanistic assumptions of the exponential model. However, because the raw Jemski data are not published, it is not possible to test whether alternate models would have provided a good fit, and it is possible that the low dose estimates of model **J** are highly extrapolated from the data points. Given this possibility, the fact that the goodness of fit for the log-probit model was not reported, and that the log-probit model does not have a defensible theoretical derivation, we feel that model **B4** is better supported for use in quantitative analyses.

Our models **D3** and **B4** also differ in key ways from previously published models fit to the same data sets. Our optimal estimate for the exponential model parameter *r* fit to the Druett data set (model **D3**) is two times higher than the value calculated by Haas [18] for the same model fit to the same data set. The lower infectivity estimated in that paper results from an estimated respiration rate of 2.4 L/min rather than the value of 1.2 L/min reported and used for calculations in the original paper [27]. Our value of *r* for model **B4** is also higher than published estimates by both Haas [18] and Mayer *et al.* [35] for models to the same data set (Figure 2). Our refinement demonstrates that it can be important to consider the possibility that apparently healthy animals sacrificed after being exposed might have become infected had they lived, especially if they were exposed to a pathogen like *B. anthracis* for which substantial incubation periods can occur.

Our model **B4** provides estimates for the distribution of the incubation period, that is, the time between exposure and the onset of symptoms. The estimate of 12 days (95% range 10–15 days) for the median incubation period for those infected by low doses (ID_10_ or less) is consistent with the 13-day median observed among autopsy-confirmed cases after the Sverdlovsk release, for which a less than 2% attack rate was estimated. The full distribution of incubation periods is important for risk planning under a large scale release scenario, as it indicates how soon after a release cases would begin appearing, the period during which the bulk of cases would appear, and how long new cases might continue to appear toward the end of the outbreak. For example, model **B4** estimates a minimum incubation period of 2.3 days (95% range 0 to 5.4 days), suggesting that no symptomatic cases would appear until at least that amount of time after an exposure event. While this estimate is primarily derived from non-human primate data, it appears to be consistent with observations of human cases. The IOM found no examples of well-documented human incubation periods less than 4 days, but there are unconfirmed reports of incubation periods as low as 2 days among Sverdlovsk cases [12]. Under a scenario similar to Sverdlovsk in which a large population is exposed to the ID_1_, model **B4** estimates that, in the absence of prophylactic treatment, the first 10% of cases would appear between 2 and 4 days after exposure, the middle half (interquartile range) of cases would appear between 6 and 22 days after exposure, and the last 10% of cases would appear over 35 days after the minimum incubation period. These estimates and their associated confidence intervals are largely consistent with the distribution of autopsy-confirmed cases after Sverdlovsk (Figure 4).

The incubation period distribution produced by our model **B4** is unique among others in the literature [12], [32], [37] in that Sverdlovsk data were not used to derive its parameter values. Nevertheless, its estimates compare quite favorably with those of the other models in capturing the distribution of autopsy-confirmed Sverdlovsk cases (Figure 4) under the assumption of ID_1_ exposure. The other models generally predict shorter incubation periods than our model, although the curves fall within our 95% confidence region over approximately the first week after exposure. The difference might be explained by the fact that the other models optimized parameter values using larger Sverdlovsk data sets that include unconfirmed cases of unusually short incubation periods, which were questioned in a recent IOM review [12].

Our analyses also provide a framework for modeling the effects of inhaling multiple doses at different times as a natural extension to the mechanistically based competing risks model [32]. This allowed us to make use of the Brachman data consisting of irregular exposures over several weeks (similar to Mayer *et al.* [35]), which had previously been modeled only using averaging techniques [18] in which the temporal information in the data were lost. The model is potentially useful for any pathogen in which chronic low-dose exposure is important.

**Figure 6:**
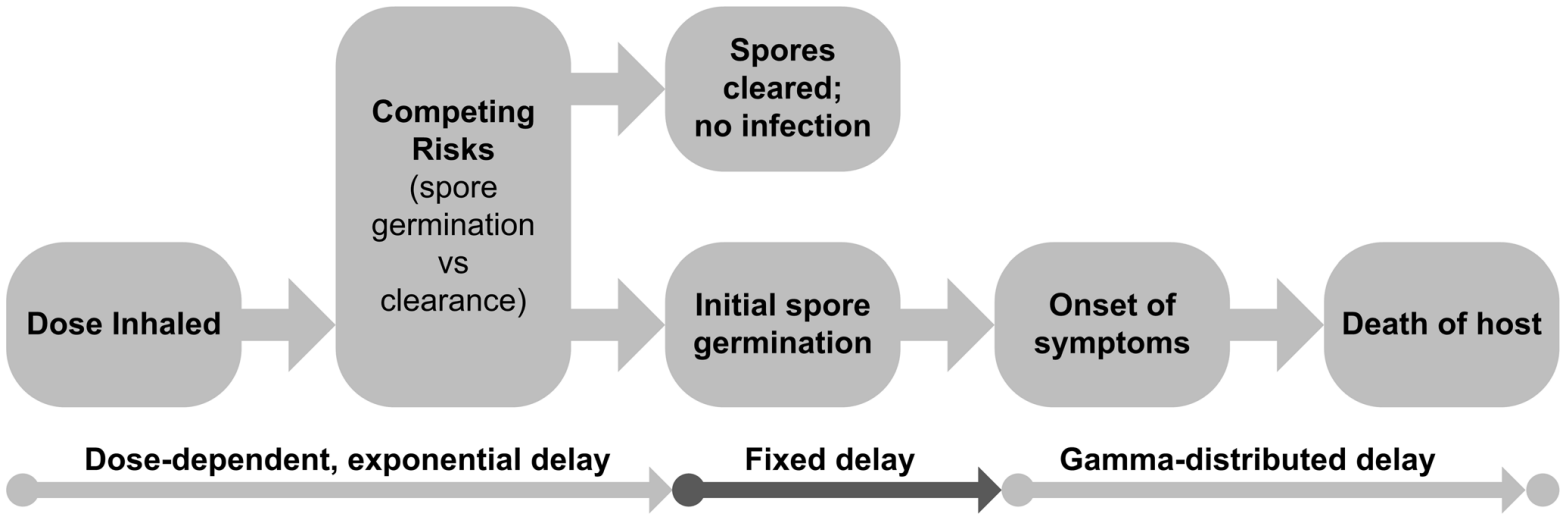
Schematic of the determination of infection and the infection timeline for anthrax. This depicts the assumptions made under the time-dose-response model **B4**, developed for this paper. After a dose of a given size is inhaled, a competing risks process determines whether infection occurs and the distribution of the time between exposure and infection (initial spore germination) if it does occur. We assume a fixed delay between initial spore germination and the onset of symptoms and a gamma-distributed delay between the onset of symptoms and death among untreated cases.

In model **B4**, we have provided a framework for modeling the time between four key moments of disease progression: exposure, infection (initial spore germination), onset of symptoms, and death (Figure 6). We designed the mathematical representation of this process both to make use of the best available data in a transparent manner and to create a parsimonious model that relies on as few free parameters as possible for adequate fitting to data.

Our choice of the spore clearance rate parameter *θ* = 0.07, which characterizes the exposure-to-infection portion of the disease progression timeline, was based on calculations from direct observation of the lungs of exposed non-human primates. Other estimates of this parameter [32], [35] were derived from model fitting procedures that relied in part on ad-hoc assumptions of other portions of disease stage timing process.

The gamma distribution we applied to quantify the time from symptom onset to death was fit to data from the best documented human cases [25]; shorter estimates derived from Sverdlovsk data may suffer from inaccurate or incomplete information from those cases [37]. Our choice of a two-parameter gamma distribution is, for our purposes, more parsimonious than the four-parameter model used by Holty *et al.* [25]. Their more complicated model has the benefit of separating the symptomatic period into distributions for prodromal and fulminant stages, although the individual-level data for the timing of transitions between these sub-stages are not provided, which limits reproducibility.

Finally, we modeled the remaining portion, the germination-to-symptoms delay, using a single-parameter fixed delay, for simplicity. Wilkening [37] used a more complicated model for this delay incorporating dose-dependency. A large dose could cause a shorter expected delay if multiple spores germinate in a short time period, thus contributing more initial vegetative spores that undergo exponential growth towards the symptoms threshold. For lower doses, the primary focus of our paper, the probability of even one spore germinating on a given day is small, and the probability of additional spores germinating in a time frame short enough to contribute substantially to the expected delay is assumed to be negligible.

As in the results of Brookmeyer *et al.* [39], our estimates of the probability of infection at 60 days post-exposure based on various inhaled doses of spores provide a defensible rationale and support for the current recommendation of a 60 day duration of prophylaxis using appropriate antimicrobials after low dose exposure scenarios. For doses close to the ID_1_ (100–250 spores, by our model **B4**), which was approximately the attack rate after the Sverdlovsk release [7] and the 2001 incident at two postal facilities and a media company [39], an antibiotic course completed 60 days post exposure reduces the probability of infection to 0.015% (about one in 7,000 chance). As illustrated in Figure 5, applying our parameter values to the Brookmeyer equation (stated in *Materials and Methods*) can shed light on the implications of higher dose exposures for the issue of prophylactic duration, as well as the implications of shortened courses due to non-adherence to recommendations, which has been an important issue historically [44] and in public health planning for potential release events [45]. Development of extended mathematical models that incorporate variable effectiveness of antibiotics, the effects of irregular adherence patterns, and balancing decreased infection probability against adverse effects of long term antibiotic exposure [46] could be an important direction for future work.

Our analysis has some limitations. In reviewing the literature, there are experimental studies of *B. anthracis* dose-response using mice, rabbits, and guinea pigs [47]–[49]. We have restricted our studies to non-human primate data. Components of our analyses and discussions based on data from and prior analyses of the Sverdlovsk release and other human data are subject to potential limitation of those data and analyses. Namely, epidemiologic data collected in retrospect may contain errors, and simplifying assumptions regarding the airborne transport of released spores at Sverdlovsk may have caused inaccurate representations of the exposure profile across the affected population. Finally, our quantitative estimates are largely based on data from non-human primates, which may have important differences from humans with regard to susceptibility and disease progression. However, the consistency of our incubation time model with the Sverdlovsk data offers compelling evidence for the plausibility of the model under human exposure scenarios.

In conclusion, we have synthesized and improved existing inhalational anthrax dose-response models to derive defensible and plausible estimates with respect to infectious doses, incubation periods, and duration of antibiotic prophylaxis needed in the event of human exposure.

## Materials and Methods

### Ethics statement

This study was reviewed by the Institutional Review Board (IRB) of the University of Utah and determined to be exempt from IRB oversight as the project does not meet the definitions of Human Subjects Research according to Federal regulations.

### Software

We used R version 3.0.0 [50] for calculations, optimization of parameter values for fitting models to data (standard functions optim and optimize), and generation of figures to display results (standard plotting functions and the gridBase package), all freely available.

### Description of dose response and incubation time models

We use or compare the following models in this paper. In the equations, *I*(*d*) is the probability of infection after inhaling dose *d*.

- Log-probit model: 
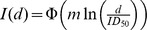

  The parameter ID_50_ refers to the dose at which there is a 50% chance of infection, and *m* is a second parameter called the probit slope. The function Φ is the cumulative distribution function for the standard normal distribution, and ln is the natural logarithm. Note that some models we cite instead used the base-10 logarithm, so their reported values of the probit slope differ from what is listed under Table 1 by a factor of ln 10. The model was first developed [51] as a convenient method for transforming experimental data into approximately linear form so that regression could more easily be done by hand. The method was popularized for use in applications to toxicology [52] and has since become the traditional model used in toxicological risk assessment. The model is still used despite the fact that its originally espoused advantage of ease of hand calculation is no longer relevant with the advancement of computer technology. Some authors (e.g., [53]) have argued that the log-probit model is not a preferred choice because it is not based on any clear assumptions about biological mechanisms for the establishment of infection. However, others have argued that the log-probit model is an appropriate model when the host population is heterogeneous (e.g., [15]); for example, if each potential host has a tolerance (a dose that is just sufficient for establishing infection), and the variation in tolerances across the population is adequately captured by the lognormal distribution, then the log-probit model may be justified [52].
- Exponential model: 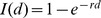

  As derived in [33], the single parameter *r* is defined as the probability that infection is established by a single organism. A single organism establishing infection means that the organism produces descendants in the host that survive to contribute to a sustained population in the host. In this sense, the exponential model is an example of a “single hit” model. The model assumes that multiple organisms act independently in the host. That is, the probability that any one organism in the initial dose produces descendents in an eventual infection is independent of the size of the dose. If the exact number of organisms in the dose is known, then the overall probability of infection is simply the complement to the probability that none of the organisms establish infection, or 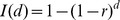. The exponential model follows after assuming that there is uncertainty in the size of the dose. Specifically, the assumption is that *d* is the expected value for the number of organisms in the dose, and the true value varies according to a Poisson distribution with mean *d*.
- Exponential model with time dependence: 
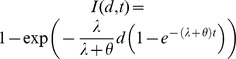

  Here, *I*(*d*, *t*) is the probability that infection occurs sometime before time *t* after exposure to a dose *d*. This version of the exponential model was derived from a “competing risks model” constructed specifically for *B. anthracis* [32], in which *λ* represents the risk per unit time that an inhaled spore germinates, and *θ* represents the risk per unit time that an inhaled spore is cleared from the lung. The model also characterizes the distribution of the time to infection (initial spore germination) after inhaling a given dose. For large values of *t*, the equation reduces to the standard exponential model with *r* = *λ*/(*λ*+*θ*). Furthermore, evidence from non-human primates shows that *λ* is on the order of 10^−4^ or less, while *θ* is on the order of 10^−1^. Therefore, the quantity *λ*+*θ*≈*θ*. Using both of these simplifications, the equation for *I*(*d*, *t*) approximates as *I*(*d*, *t*) = 1−exp(−*rd*(1−e^−*θt*^)).
- Beta Poisson model: 
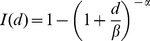

  Here, *α* and *β* are constant parameters. Like the exponential model, the traditional derivation of the beta Poisson model [54] begins with the assumption of a “single-hit” framework where organisms act independently. Again, under these assumptions, if the exact dose *d* is known and if the probability *r* of a single organism establishing infection is constant, the probability of infection would be given as 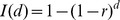. The beta Poisson model, like the exponential model, assumes that the dose *d* is a random Poisson-distributed variable with mean *d*, and additionally assumes that the probability *r* varies according to a beta distribution with parameters *α* and *β*. These assumptions alone lead to a dose-response model with a complicated functional form involving the Kummer confluent hypergeometric function (see [33]). The simpler beta Poisson formula above is an approximation to the exact formula, and the approximation is valid when individual probabilities are low, or *β*>>1 and *β*>>*α*. With this approximation, the beta Poisson model is not a single-hit model [55]. The beta Poisson model can also be derived from the assumption that the dose varies according to a negative binomial distribution, which allows for a more highly variable distribution of doses than under the simpler Poisson assumption [33].
- Exponential model with Mayer *et al.* [35] extension: 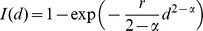

  Mayer *et al.* [35] extended the exponential model described above to account for the possibility that the per capita probability of infection increases with increasing single dose received, under the assumption that the immune system of the host would be more likely to be overwhelmed by a higher single dose. The parameter *α*, constrained by assumption to be less than or equal to one, is a shaping parameter that quantifies the effect of the size of a single dose on the per-spore clearance rate. The parameter *r* is roughly equivalent to the same parameter in the basic exponential model and is exactly equivalent when *α* = 1, for which this model reduces to the exponential model. The parameter *α* in itself does not have a well defined biological meaning; however, a result showing that dose-response data support a value of *α* significantly less than one would suggest immune system effects related to dose size as a potentially important mechanism for further investigation. A time-dependent version of this model is presented in [35].
- Age-dependent logit model: 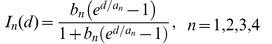

  This model was defined by Webb and Blaser [16], with each of the *a_n_* and *b_n_* parameters values derived from ID_10_ and ID_50_ values assumed for four different age ranges, with individuals in younger age brackets being less susceptible to infection, as follows: *a*_1_ = 6.5×10^3^; *a*_2_ = 4.4×10^3^; *a*_3_ = 2.6×10^3^; *a*_4_ = 6.5×10^2^; and *b_n_* = 0.11 for each *n*. The age ranges are <25, 25–44, 45–65, and >65 for *n* = 1, 2, 3, and 4, respectively. Logit models are commonly used as generalized linear models in a variety of scientific fields. Like the log-probit model, the logit model does not have a known derivation based on assumed biological mechanisms of infection.
- Age-dependent linear model: 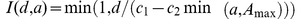

  This model, defined by Craft *et al.* [10], assumes that the probability of infection for a given individual increases linearly with the dose inhaled, up to a dose above which infection is certain (probability one), and that the slope of this linear function varies with the age, *a*, of the individual. The parameter values are based on the values provided by Webb and Blaser [16], as follows: *c*_1_ = 38,000; *c*_2_ = 450; and *A*_max_ = 80.
- Age-dependent probit model: 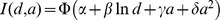

  This model, defined by Wein *et al.* [9], is equivalent to the log-probit model defined above, except for the dependence on the age, *a*, of the exposed individual. The age variable coefficients were derived from the values provided by Webb and Blaser [16], as follows: *α* = −9.733; *β* = 1.025; *γ* = −0.016; and *δ* = 0.0006.
- IOM model for incubation period distribution: 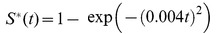

  Here, *S* ^*^(*t*) is a dose-independent cumulative distribution function for the probability that the incubation period (time between exposure and onset of symptoms among those infected) would be less than *t* hours. The equation was suggested by the IOM [12] for use in models of the distribution of the incubation period of anthrax over approximately the first eight days after exposure of a population to an unspecified dose, for purposes of comparing the effects of potential post-exposure intervention strategies.
- Brookmeyer incubation period distribution: 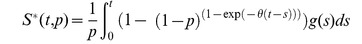

  As defined in Brookmeyer *et al.* [32], the value *p* refers to the probability of infection under the exponential dose-response model at a given dose; i.e., *p* = 1−exp(−*rd*), where *d* is the inhaled dose and *r* is the exponential model parameter, defined previously. This, therefore, defines a dose-dependent incubation period distribution. The parameter *θ* is the spore clearance rate from the time-dependent exponential model, defined previously. The function *g*(*s*) is the density function of the distribution for the time between initial spore germination and the onset of symptoms. They applied the exponential distribution, for which *g*(*s*) = *γ* exp(−*γs*). The parameter *γ*, often called the rate parameter, governs the median of the distribution. In this case, the authors' proposed best model assumed a median time of 2 days for this delay, so that *γ* = (ln 2)/2≈0.347 per day. Under this assumption, and also under the assumption of a low dose of exposure (small *p*), they optimized *θ* against data from human incubation periods from the Sverdlovsk release, finding *θ* = 0.109. Wilkening [37] extended this model by assuming a lognormal distribution for the function *g*(*s*), with the median of this distribution being dependent on the dose of exposure and other parameters.

### Criteria for evaluating dose-response models for human exposure to *B. anthracis*

We developed objective criteria to evaluate candidate dose-response models for human inhalational anthrax. The criteria were informed by a review of the literature, a critical evaluation of existing dose-response models for strengths/weaknesses, and discussion with members of our research team consisting of professional risk assessors, mathematical modelers, microbiologists, veterinarians, and infectious disease physicians and epidemiologists. Precedence for developing such criteria exist in the field of biodefense, specifically with regard to applying mathematical and simulation modeling to inform public health action and policy [56]. The four criteria are:

1. **The parameter values are derived from dose-response data.** The model should be fit to documented, quantitative data, so that the strengths and limitations of those data and their effects on the model estimates are transparent.
2. **The shape of the dose-response curve is consistent with Sverdlovsk data.** Given the analysis in [15], the temporal and spatial distribution of cases observed after the Sverdlovsk release provide evidence for the shape of dose-response curves that are appropriate for application to human exposure. Thus, a plausible *B. anthracis* dose-response model should be consistent with that evidence.
3. **The model is derived from mechanistic assumptions.** Mathematical models derived from biological or physical assumptions provide insight into the processes that govern the establishment of infection. Scientific advances in the understanding of those processes can serve to improve the models.
4. **The model estimates the incubation period.** A model that can produce both estimates of infection probability and of the incubation period at a given dose is more useful than one that provides only estimates of infection probability, as the time from exposure to infection has important implications for intervention and mitigation strategies after a release of anthrax spores in a community.

### Model fitting to Druett *et al.* data

For fitting to the Druett at al. [27] data, shown in Table S1, we use three different mathematical forms for the function *I*(*d*), the probability of infection if dose *d* is received: the log-probit model, the exponential model, and the beta Poisson model. We obtain the parameter values of these models using maximum likelihood estimation employing the binomial distribution [33]. Under this method, the optimal parameter values minimize the deviance, *Y*, defined as
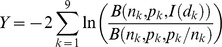



In this formula, *k* is the number of the experimental group (there were nine different groups that each received different levels of exposure), *n_k_* is the number of animals exposed in group *k*, *p_k_* is the number of positive responses (anthrax deaths) in group *k*, and *d_k_* is the dose received by the animals in group *k*. The function *B*(*n*, *p*, *q*) is the probability mass function for the binomial distribution, which represents the probability of *p* successes in *n* trials when the probability of success in each individual trial is *q*. In essence, the parameters embedded in the *I*(*d*) function are being optimized so that the formula matches as closely as possible the infection rates, *p_k_*/*n_k_*, observed in the exposure groups.

### Model fitting to Brachman *et al.* data

The data derived from the three experimental runs of Brachman *et al.* [24], shown in Tables S2, S3, S4, describe the doses received on each day, the days of death due to anthrax or other causes, and the number of animals found to be infected upon sacrifice. To fit these data using a time-dependent model, a conceptual framework is required for the time course of anthrax infection from exposure to death. We develop such a framework, which differs from the framework developed in [35], as follows.

The model represents three main stages of the anthrax infection timeline in humans after inhalational exposure (Figure 6), as follows, in preparation for fitting to the Brachman *et al.* data set.

#### 1. Competing risks of spore germination and clearance

We use the exponential model with time dependence [32], under mechanistic assumptions regarding competing risks of spore germination and clearance as described above. Infection is defined as the germination of at least one of the spores inhaled; no infection occurs if all the spores are cleared before germination. We use the equation form
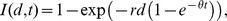
derived above, in data fitting procedures in this paper. This framework allows one to calculate, given the size of the initial dose, both the probability that infection eventually will occur and the probability distribution of the time between exposure and initial spore germination, if it occurs.

#### 2. Delay between initial spore germination and the development of symptoms

While signs of infection in the animals prior to death were not recorded in the Brachman *et al.* experiments, it is conceptually important for our model, when applied to humans, to include the delay between initial spore germination and the onset of symptoms (incubation period) as a subset of the overall delay between infection and death. Studies of the *B. anthracis* infection process have shown that symptom onset occurs some time after initial spore germination [37]. The delay is due to the stages of bacterial growth, which include a lag phase, an exponential growth stage, a stationary phase once the bacterial population reaches a threshold, and a decline phase during which toxins produced by *B. anthracis* build up in the host. Symptoms are presumed to occur during the latter two stages, which correspond roughly to the prodromal and fulminant phases of disease in the host. Because no direct data on this delay exist, we incorporate the delay as a fixed parameter *T*, to be optimized in light of the overall data under the assumption of given distributions for the exposure-to-germination and symptoms-to-death delays, for which data are available from other sources. The parameter *T* can be interpreted as the time required for an exponentially growing cell population, starting from a single cell, to grow to a threshold size at which symptoms would begin appearing. If the doubling time is *t*_2_ days, and the threshold population size is *N*, then *T* = *t*_2_ ln *N*/ln 2. Under this assumption, *S*(*d*, *t*), the expected fraction of individuals that would exhibit symptoms in less than *t* days after inhaling a dose of *d* spores of *B. anthracis*, is defined as follows.
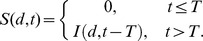



#### 3. Delay between onset of symptoms and death

We use this final delay to complete the overall model of the delay between exposure and death, which was recorded for non-human primates in the Brachman *et al.* data. Again, the times of the first signs of infection were not reported in the Brachman data, but human data are available for 82 historical infections [25] that can be used to create a model for this part of the overall delay time course. To do this, we used a simplified version of the model fitting procedure used by Holty *et al.* [25], described as follows.

We are interested in a distribution for the time between the onset of symptoms and death among untreated individuals. Among the 82 cases in the human data set, 38 were cases in which no appropriate treatment was received, and the time to death was available. Fitting a model to only those 38 cases is an option, but this approach could introduce a bias due to censoring. Individuals with symptoms that worsened more slowly than average probably had a better chance of receiving antibiotic treatment before death and were therefore more likely not to be included in those 38 cases. To include information from the cases who received antibiotic treatment, we made the following assumptions. For those who received appropriate treatment and still died (26 cases), we assume those data are *interval censored*, meaning that the time of death in the absence of treatment would have occurred sometime in the interval between the time that treatment began and the time that death occurred (i.e., the treatment did not prevent death but may have delayed it). For those who received appropriate antibiotic treatment and survived (11 cases), we assume those data are *right censored*, meaning that they would have died if they had not received treatment and the time of death would have been some time after the time that treatment began. We exclude one case of a survivor who was thought to have been partially immune due to prior exposure as a veterinarian, and we exclude six untreated cases who died but no information was given on the time of death.

We fit the exponential distribution, the gamma distribution, the Weibull distribution, and the lognormal distribution to these data using the method of maximum likelihood, defining the log likelihood as follows:
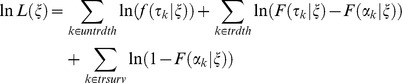



In this formula, the natural logarithm of the likelihood, *L*, is a function of the parameters of the model being tested, represented as *ξ*. The human anthrax case data are split into three categories as described above, denoted in the equation as untreated deaths (*untrdth*), those who died without receiving appropriate antibiotic treatment, treated deaths (*trdth*), those who died after receiving antibiotic treatment, and treated survivors (*trsurv*), those who survived after receiving antibiotic treatment. The data consist of *τ_k_*, the time of death of case *k*, if applicable, and *α_k_*, the time that antibiotic treatment began for case *k*, if applicable. The functions *f* and *F* are the probability density function and the cumulative distribution function, respectively, of the distribution being tested.

Of the four distributions tested, the gamma distribution provided the greatest likelihood under its optimal parameter values and was chosen for use in our overall model. The best fit gamma distribution has probability density function
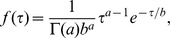
with shape parameter *a* = 5.43 and scale parameter *b* = 0.864. This distribution results in an average time of 4.7 days, with a standard deviation of 2.0 days. The mortality function *M*(*d*, *t*) represents the fraction of individuals that would die in less than *t* days after inhaling a dose of *d* spores.
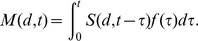



The formulas above for *I*(*d*, *t*), *S*(*d*, *t*), and *M*(*d*, *t*) apply in continuous time under a scenario in which a single dose is inhaled at time zero, with no subsequent exposure at later times. Those formulas require modification to model the Brachman *et al.* data, which consist of multiple exposures across several days. The modeling framework described above is naturally extended to the multiple dose scenario, because the models provide estimates for the number of spores retained in the lung from previous exposures at times when new exposures are experienced. Thus, we can obtain formulas for the cumulative number of spores in the lung across multiple days of exposure and the risk of infection associated with the number of spores in the lung at a given time. Mayer *et al.* [35] used this strategy to derive models that can be fit to the Brachman data. We define our own formulation as follows.

In the following formulas, the dose input paired with time *t* is given as a vector *d*_<*t*_ = (*d*_1_, *d*_2_, … , *d_t_*_−1_), where *d_τ_* is the dose received on day *τ*. The initial exposure is defined as occurring on day 1, and it is assumed that the probability of infection or death on day *t* is affected by the doses inhaled from day 1 to day *t*−1, which means that day 2 is the first day that infection is possible under the model. These formulas are for the probability of infection (*I*), symptoms onset (*S*), and mortality (*M*) occurring before day *t* of the experimental run.
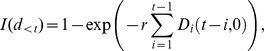


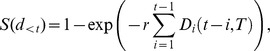


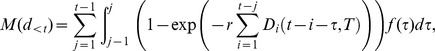
where
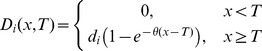
represents the contribution of the dose inhaled on day *i* to the risk of a response occurring by time *x*.

This model contains five biological parameters. Based on a result in (32), we fix *θ*, the probability-per-time for clearance of spores from the lung, at 0.07 per day, and we fix the parameters *a* and *b* from the *f*(*τ*) function as described above. The remaining two parameters are *r*, the probability of each spore successfully germinating before being cleared, and *T*, the delay between initial spore germination and onset of symptoms. We optimize these two parameters together in light of the Brachman *et al.* data using maximum likelihood estimation, employing the binomial distribution and treating cumulative information from each day of the Brachman experiments as separate data points, similar to the method used in [57]. That is, we minimize the deviance, *Y*, defined as follows.
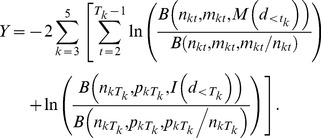



In this formula, *k* is the number of the experimental run (we used Brachman runs 3, 4, and 5, shown in Tables S2, S3, S4), *T_k_* is the total number of days in run *k*, *n_kt_* is the number of subjects relevant for the model on day *t* of run *k* (all subjects minus those having died of a non-anthrax cause prior to that day), *m_kt_* is the cumulative number of deaths due to anthrax on or before day *t* in run *k*, and *p_kt_* is the cumulative number of anthrax infections, which is known on the last day, *T_k_*, of each run when the remaining animals were sacrificed and examined for infection. The function *B*(*n*, *m*, *q*) is the probability mass function for the binomial distribution, which represents the probability of *m* successes in *n* trials when the probability of success in each individual trial is *q*.

The functions *M* and *I* contain the dose-response parameter values, which we optimize by minimizing the deviance *Y*. For each model, we compare the deviance to the upper 5^th^ percentile of the χ^2^ distribution with degrees of freedom equal to the number of distinct dose-time pairs in the data set minus the number of parameters being optimized. We reject a null hypothesis of fit acceptability if the optimal deviance is greater than the corresponding χ^2^ statistic [33]. We calculate the confidence intervals for these two parameters and for infectious dose estimates by fitting the model to bootstrap resamples of the Brachman data.

### Dose-response and time course estimates after exposure to a single dose

The best fit model to the Brachman data produces an estimate for the time-independent dose-response curve, which is simply the regular exponential model: *I*(*d*) = 1−e^−*rd*^. We use our result for the optimized parameter *r* and associated confidence interval for comparing our result to other dose-response curves used in the literature. We also display our model's estimates of the incubation period (time between exposure to symptom onset among those infected) distribution *S* ^*^(*d*, *t*), which is the probability of symptoms appearing by time *t* after a single dose *d*, *S*(*d, t*), divided by the probability that infection occurs at any time, *I*(*d*). I.e.,
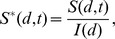
or
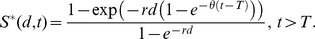



### Probability of infection after prophylaxis

We apply our best fit model parameters to the equation developed in [39] for calculating the effect of various durations of antibiotic use on reducing the probability of infection, if prophylactic medications were administered after exposure. If an individual is exposed to a dose *d* and adheres to an effective prophylactic regimen for *τ* days after exposure, the probability *Q*(*τ*) that infection occurs after the prophylactic regimen ends is given by
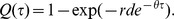



This equation assumes that i) spores cannot germinate successfully during the antibiotic course, ii) antibiotics do not affect the clearance rate of spores that have not germinated, and iii) spores germinating after the antibiotic course is finished would cause infection.

The views expressed in this paper are those of the authors and do not necessarily represent the views of the Department of Veterans Affairs or the United States Government.

## Supporting Information

Figure S1 **Sensitivity plots for model B4.** In all plots, solid line is produced by model **B4** under the assumption of ID_1_ exposure. Dashed lines in A and B show sensitivity of the cumulative distribution function to changing the assumption to ID_50_ exposure and to assuming *θ* = 0.11 instead of *θ* = 0.07. C and D show sensitivity of the probability density function to the same changes. Points in A and B and histogram in C and D are from autopsy-confirmed anthrax deaths after the Sverdlovsk release (see main text).
(EPS)

Table S1 **Non-human primate inhalational anthrax dose-response data from Druett** ***et al.*** [**27**] **.** We calculate doses above as the product of the following values reported in [27]: air concentration of exposure (spores per L), breathing rate of 1.2 L/min, and exposure of time of 1 min.
(DOC)

Table S2 **Data from Brachman** ***et al.*** **** [**24**] **Run 3: 32 monkeys.** We recorded dose data from text in [24] where available. Otherwise, we visually estimated the daily doses from Figure 3 therein. ^a^Two sacrificed animals found to be infected with anthrax on day 50. ^b^We consider data from days of sacrifice to be number of animals *infected* by that day.
(DOC)

Table S3 **Data from Brachman** ***et al.*** **** [**24**] **Run 4: 31 monkeys.** We recorded dose data from text in [24] where available. Otherwise, we visually estimated the daily doses from Figure 3 therein. ^a^We consider data from day of sacrifice to be number of animals *infected* by that day.
(DOC)

Table S4 **Data from Brachman** ***et al.*** **** [**24**] **Run 5: 28 monkeys.** We recorded dose data from text in [24] where available. Otherwise, we visually estimated the daily doses from Figure 3 therein. ^a^We consider data from the day of sacrifice to be number of animals *infected* by that day.
(DOC)

Table S5 **Cumulative data from Brachman** ***et al.*** **** [**24**] **.** Haas [18] fit the exponential dose-response model without a time component to the Brachman data, using an averaging technique [28] that is equivalent to applying the total cumulative dose over each Brachman experimental run as a single data point, as if that cumulative dose was a one-time exposure. The actual total cumulative doses from Brachman runs 3 and 4 as reported were lower that what was applied by Haas. The error was caused by multiplying the reported average daily exposure by the number of days between the first and last exposures, rather than the number of days on which exposure actually occurred.
(DOC)
