# Supplementary material for: Quantitative Models of the Dose-Response and Time Course of Inhalational Anthrax in Humans
Source: PLoS Pathog. 2013 Aug 15;9(8):e1003555. doi: 10.1371/journal.ppat.1003555 (PMC3744436; doi:10.1371/journal.ppat.1003555)
Supplement: Table S1 — Non-human primate inhalational anthrax dose-response data from Druett et al. [27] . We calculate doses above as the product of the following values reported in [27]: air concentration of exposure (spores per L), breathing rate of 1.2 L/min, and exposure of time of 1 min. (DOC) [file ppat.1003555.s002.doc]

**Table S1. Non-human Primate Inhalational Anthrax Dose-Response Data from Druett *et al*.**

| **Dose (spores)** | **Number exposed** | **Number died** |
| --- | --- | --- |
| 35,160 | 8 | 1 |
| 38,520 | 8 | 4 |
| 54,360 | 8 | 5 |
| 68,760 | 8 | 6 |
| 77,760 | 8 | 5 |
| 80,400 | 8 | 3 |
| 120,000 | 8 | 8 |
| 150,000 | 8 | 7 |
| 199,200 | 8 | 8 |

We calculate doses above as the product of the following values reported in : air concentration of exposure (spores per L), breathing rate of 1.2 L/min, and exposure of time of 1 min.
