# Supplementary material for: Quantitative Models of the Dose-Response and Time Course of Inhalational Anthrax in Humans
Source: PLoS Pathog. 2013 Aug 15;9(8):e1003555. doi: 10.1371/journal.ppat.1003555 (PMC3744436; doi:10.1371/journal.ppat.1003555)
Supplement: Table S2 — Data from Brachman et al. [24] Run 3: 32 monkeys. We recorded dose data from text in [24] where available. Otherwise, we visually estimated the daily doses from Figure 3 therein. aTwo sacrificed animals found to be infected with anthrax on day 50. bWe consider data from days of sacrifice to be number of animals infected by that day. (DOC) [file ppat.1003555.s003.doc]

**Table S2. Data from Brachman *et al*. Run 3: 32 monkeys**

We recorded dose data from text in where available. Otherwise, we visually estimated the daily doses from Figure 3 therein. ^a^Two sacrificed animals found to be infected with anthrax on day 50. ^b^We consider data from days of sacrifice to be number of animals *infected* by that day.

| **Day** | **Dose (spores)** | **Anthrax deaths** | **Other cause deaths** | **Cumulative anthrax deaths / Number possible** |
| --- | --- | --- | --- | --- |
| 1 | 2,500 | - | - | 0 / 32 |
| 2 | 1,625 | - | - | 0 / 32 |
| 3 | 375 | - | - | 0 / 32 |
| 4 | 800 | - | - | 0 / 32 |
| 5 | 385 | - | - | 0 / 32 |
| 6 | 0 | - | - | 0 / 32 |
| 7 | 0 | - | - | 0 / 32 |
| 8 | 0 | - | - | 0 / 32 |
| 9 | 450 | - | 1 | 0 / 32 |
| 10 | 32 | - | - | 0 / 31 |
| 11 | 710 | - | - | 0 / 31 |
| 12 | 150 | - | - | 0 / 31 |
| 13 | 0 | - | - | 0 / 31 |
| 14 | 0 | - | - | 0 / 31 |
| 15 | 55 | 2 | - | 2 / 31 |
| 16 | 225 | 1 | - | 3 / 31 |
| 17 | 150 | 1 | - | 4 / 31 |
| 18 | 350 | 2 | - | 6 / 31 |
| 19 | 470 | - | - | 6 / 31 |
| 20 | 0 | - | - | 6 / 31 |
| 21 | 0 | - | - | 6 / 31 |
| 22 | 610 | - | - | 6 / 31 |
| 23 | 870 | - | - | 6 / 31 |
| 24 | 20 | 1 | - | 7 / 31 |
| 25 | 60 | - | - | 7 / 31 |
| 26 | 510 | 1 | - | 8 / 31 |
| 27 | 0 | - | - | 8 / 31 |
| 28 | 0 | - | - | 8 / 31 |
| 29 | 710 | - | - | 8 / 31 |
| 30 | 110 | - | - | 8 / 31 |
| 31 | 950 | 1 | - | 9 / 31 |
| 32 | 450 | - | - | 9 / 31 |
| 33 | 350 | - | - | 9 / 31 |
| 34 | 0 | - | - | 9 / 31 |
| 35 | 0 | - | - | 9 / 31 |
| 36 | 1,500 | - | - | 9 / 31 |
| 37 | 225 | - | - | 9 / 31 |
| 38 | 800 | - | - | 9 / 31 |
| 39 | 575 | - | - | 9 / 31 |
| 40 | 425 | - | - | 9 / 31 |
| 41 | 0 | 1 | - | 10 / 31 |
| 42 | 0 | - | - | 10 / 31 |
| 43 | 0 | - | - | 10 / 31 |
| 44 | 225 | - | - | 10 / 31 |
| 45 | 295 | 1 | 1 | 11 / 31 |
| 46 | 0 | 1 | - | 12 / 30 |
| 47 | 0 | - | - | 12 / 30 |
| 48 | 0 | - | - | 12 / 30 |
| 49 | 0 | - | - | 12 / 30 |
| 50 | 0 | 2^a^ | - | 14^b^ / 30 |
| 51 | 0 | - | - | 14^b^ / 30 |
