# Supplementary material for: Quantitative Models of the Dose-Response and Time Course of Inhalational Anthrax in Humans
Source: PLoS Pathog. 2013 Aug 15;9(8):e1003555. doi: 10.1371/journal.ppat.1003555 (PMC3744436; doi:10.1371/journal.ppat.1003555)
Supplement: Table S4 — Data from Brachman et al. [24] Run 5: 28 monkeys. We recorded dose data from text in [24] where available. Otherwise, we visually estimated the daily doses from Figure 3 therein. aWe consider data from the day of sacrifice to be number of animals infected by that day. (DOC) [file ppat.1003555.s005.doc]

**Table S4. Data from Brachman *et al*. Run 5: 28 monkeys.**

We recorded dose data from text in where available. Otherwise, we visually estimated the daily doses from Figure 3 therein. ^a^We consider data from the day of sacrifice to be number of animals *infected* by that day.

| **Day** | **Dose**  **(spores)** | **Anthrax deaths** | **Other cause deaths** | **Cumulative anthrax deaths / Number possible** |
| --- | --- | --- | --- | --- |
| 1 | 212 | - | - | 0 / 28 |
| 2 | 232 | - | - | 0 / 28 |
| 3 | 503 | - | - | 0 / 28 |
| 4 | 0 | - | - | 0 / 28 |
| 5 | 0 | - | 1 | 0 / 28 |
| 6 | 0 | - | 1 | 0 / 27 |
| 7 | 0 | - | - | 0 / 26 |
| 8 | 0 | - | - | 0 / 26 |
| 9 | 0 | - | 1 | 0 / 26 |
| 10 | 0 | 1 | - | 1 / 25 |
| 11 | 0 | - | - | 1 / 25 |
| 12 | 0 | - | - | 1 / 25 |
| 13 | 0 | - | - | 1 / 25 |
| 14 | 0 | - | - | 1 / 25 |
| 15 | 0 | - | - | 1 / 25 |
| 16 | 0 | - | - | 1 / 25 |
| 17 | 0 | 1 | 1 | 2 / 25 |
| 18 | 0 | - | - | 2 / 24 |
| 19 | 0 | - | - | 2 / 24 |
| 20 | 0 | - | - | 2 / 24 |
| 21 | 0 | - | - | 2 / 24 |
| 22 | 0 | - | - | 2 / 24 |
| 23 | 0 | - | - | 2 / 24 |
| 24 | 0 | - | - | 2 / 24 |
| 25 | 0 | - | - | 2 / 24 |
| 26 | 0 | - | - | 2 / 24 |
| 27 | 0 | - | - | 2 / 24 |
| 28 | 0 | - | - | 2 / 24 |
| 29 | 773 | - | - | 2 / 24 |
| 30 | 574 | - | - | 2 / 24 |
| 31 | 0 | - | - | 2 / 24 |
| 32 | 0 | - | - | 2 / 24 |
| 33 | 0 | - | - | 2 / 24 |
| 34 | 0 | - | - | 2 / 24 |
| 35 | 0 | - | - | 2 / 24 |
| 36 | 0 | - | - | 2 / 24 |
| 37 | 0 | - | - | 2 / 24 |
| 38 | 0 | - | - | 2 / 24 |
| 39 | 0 | - | - | 2^a^ / 24 |
