# Supplementary material for: Quantitative Models of the Dose-Response and Time Course of Inhalational Anthrax in Humans
Source: PLoS Pathog. 2013 Aug 15;9(8):e1003555. doi: 10.1371/journal.ppat.1003555 (PMC3744436; doi:10.1371/journal.ppat.1003555)
Supplement: Table S5 — Cumulative data from Brachman et al. [24] . Haas [18] fit the exponential dose-response model without a time component to the Brachman data, using an averaging technique [28] that is equivalent to applying the total cumulative dose over each Brachman experimental run as a single data point, as if that cumulative dose was a one-time exposure. The actual total cumulative doses from Brachman runs 3 and 4 as reported were lower that what was applied by Haas. The error was caused by multiplying the reported average daily exposure by the number of days between the first and last exposures, rather than the number of days on which exposure actually occurred. (DOC) [file ppat.1003555.s006.doc]

**Table S5. Cumulative Data from Brachman *et al*. .**

| **Experimental run** | **Cumulative total dose, used in** | **Cumulative total dose, reported in** | **Number exposed** | **Number infected** |
| --- | --- | --- | --- | --- |
| 3 | 24,910 | 16,962 | 32 | 14 |
| 4 | 8,118 | 4,949 | 31 | 7 |
| 5a | 946 | 947 | 28 | 2 |
| 5b | 1,345 | 1,347 | 22 | 0 |

Haas fit the exponential dose-response model without a time component to the Brachman data, using an averaging technique that is equivalent to applying the total cumulative dose over each Brachman experimental run as a single data point, as if that cumulative dose was a one-time exposure. The actual total cumulative doses from Brachman runs 3 and 4 as reported were lower that what was applied by Haas. The error was caused by multiplying the reported average daily exposure by the number of days between the first and last exposures, rather than the number of days on which exposure actually occurred.
